# HIV treatment eligibility expansion and timely antiretroviral treatment initiation following enrollment in HIV care: A metaregression analysis of programmatic data from 22 countries

**DOI:** 10.1371/journal.pmed.1002534

**Published:** 2018-03-23

**Authors:** Olga Tymejczyk, Ellen Brazier, Constantin Yiannoutsos, Kara Wools-Kaloustian, Keri Althoff, Brenda Crabtree-Ramírez, Kinh Van Nguyen, Elizabeth Zaniewski, Francois Dabis, Jean d'Amour Sinayobye, Nanina Anderegg, Nathan Ford, Radhika Wikramanayake, Denis Nash, IeDEA Collaboration

**Affiliations:** 1 Institute for Implementation Science in Population Health, City University of New York, New York, New York, United States of America; 2 Graduate School of Public Health and Health Policy, City University of New York, New York, New York, United States of America; 3 R.M. Fairbanks School of Public Health, Indiana University, Indianapolis, Indiana, United States of America; 4 Indiana University School of Medicine, Indianapolis, Indiana, United States of America; 5 Johns Hopkins Bloomberg School of Public Health, Baltimore, Maryland, United States of America; 6 Instituto Nacional de Ciencias Médicas y Nutrición Salvador Zubirán, Mexico City, Mexico; 7 National Hospital of Tropical Diseases, Hanoi, Vietnam; 8 Institute of Social and Preventive Medicine, University of Bern, Bern, Switzerland; 9 Bordeaux School of Public Health, Bordeaux, France; 10 Rwanda Military Hospital, Kigali, Rwanda; 11 World Health Organization, Geneva, Switzerland; University of Southampton, UNITED KINGDOM

## Abstract

**Background:**

The effect of antiretroviral treatment (ART) eligibility expansions on patient outcomes, including rates of timely ART initiation among those enrolling in care, has not been assessed on a large scale. In addition, it is not known whether ART eligibility expansions may lead to “crowding out” of sicker patients.

**Methods and findings:**

We examined changes in timely ART initiation (within 6 months) at the original site of HIV care enrollment after ART eligibility expansions among 284,740 adult ART-naïve patients at 171 International Epidemiology Databases to Evaluate AIDS (IeDEA) network sites in 22 countries where national policies expanding ART eligibility were introduced between 2007 and 2015. Half of the sites included in this analysis were from Southern Africa, one-third were from East Africa, and the remainder were from the Asia-Pacific, Central Africa, North America, and South and Central America regions. The median age of patients enrolling in care at contributing sites was 33.5 years, and the median percentage of female patients at these clinics was 62.5%. We assessed the 6-month cumulative incidence of timely ART initiation (CI-ART) before and after major expansions of ART eligibility (i.e., expansion to treat persons with CD4 ≤ 350 cells/μL [145 sites in 22 countries] and CD4 ≤ 500 cells/μL [152 sites in 15 countries]). Random effects metaregression models were used to estimate absolute changes in CI-ART at each site before and after guideline expansion. The crude pooled estimate of change in CI-ART was 4.3 percentage points (95% confidence interval [CI] 2.6 to 6.1) after ART eligibility expansion to CD4 ≤ 350, from a baseline median CI-ART of 53%; and 15.9 percentage points (pp) (95% CI 14.3 to 17.4) after ART eligibility expansion to CD4 ≤ 500, from a baseline median CI-ART of 57%. The largest increases in CI-ART were observed among those newly eligible for treatment (18.2 pp after expansion to CD4 ≤ 350 and 47.4 pp after expansion to CD4 ≤ 500), with no change or small increases among those eligible under prior guidelines (CD4 ≤ 350: −0.6 pp, 95% CI −2.0 to 0.7 pp; CD4 ≤ 500: 4.9 pp, 95% CI 3.3 to 6.5 pp). For ART eligibility expansion to CD4 ≤ 500, changes in CI-ART were largest among younger patients (16–24 years: 21.5 pp, 95% CI 18.9 to 24.2 pp). Key limitations include the lack of a counterfactual and difficulty accounting for secular outcome trends, due to universal exposure to guideline changes in each country.

**Conclusions:**

These findings underscore the potential of ART eligibility expansion to improve the timeliness of ART initiation globally, particularly for young adults.

## Abstract 2

In a large-scale analysis, Olga Tymejczyk and colleagues study antiretroviral therapy initiation in people with HIV infection accompanying secular changes in treatment eligibility.

## Author summary

**Why was this study done?:**

- In 2009 and 2013, the World Health Organization (WHO) recommended that HIV patients with CD4 counts ≤350 and ≤500 cells/μL, respectively, initiate antiretroviral treatment (ART).
- The expansion of ART eligibility criteria has the potential to increase ART initiation rates, especially among healthier patients; however, it could also lead to “crowding out” of persons with more advanced disease and lower rates of ART initiation among these patients.
- While many countries have adopted WHO guidelines, the impact of ART eligibility expansions on timely ART initiation has not been studied on a large scale.

**What did the researchers do and find?:**

- We examined the changes in timely ART initiation after national ART eligibility criteria were expanded to CD4 ≤ 350 and/or CD4 ≤ 500 in 22 countries, using data on 284,740 adult ART-naïve patients at 171 sites in the International Epidemiology Databases to Evaluate AIDS (IeDEA) network.
- Site-level cumulative incidence of ART initiation (CI-ART) within 6 months of enrollment increased by 4.3 percentage points after national ART eligibility expansion to CD4 ≤ 350 and by 15.9 percentage points after expansion to CD4 ≤ 500.
- At the individual level, increases were greatest among patients 16–24 years old at enrollment and those newly eligible for ART. No change or small improvements in CI-ART were also observed among patients already eligible for ART before eligibility expansion.
- At the site level, sites with the lowest initial levels of CI-ART experienced the greatest increases following guideline expansions.

**What do these findings mean?:**

- Overall, ART eligibility expansions were followed by appreciable improvements in timely ART initiation.
- Many clinics can support ART initiation among newly eligible patients with less advanced disease without negatively affecting ART initiation rates among those with more advanced disease.
- These findings illustrate the potential of ART eligibility expansion to improve the timeliness of ART initiation and patient outcomes along the care cascade globally, particularly for younger adults, in support of the Joint United Nations Programme on HIV/AIDS (UNAIDS) 90-90-90 targets, thereby reducing morbidity, mortality, and onward HIV transmission.

## Introduction

Individual and population-level benefits of antiretroviral treatment (ART) are maximized when treatment is initiated soon after HIV infection occurs [1–5]. ART slows the progression of HIV to AIDS and reduces infections and mortality among those living with HIV [6,7]. In addition, ART lowers viral load in HIV-infected individuals, thereby reducing the risk of onward HIV transmission [2,7–11]. Recent studies have shown that immediate versus delayed initiation of ART reduces risks of AIDS and severe opportunistic illnesses [12]. Some studies suggest that earlier initiation of ART may also improve rates of retention in care and medication adherence, and promote more rapid achievement of viral load suppression [13–15]. As a result of this evidence, the World Health Organization (WHO) now recommends immediate initiation of ART for all people diagnosed with HIV, regardless of CD4 count [16].

A systematic review of retention in HIV care between testing and treatment among patients who were ineligible to initiate ART at the time of diagnosis estimated that less than one-third of patients remained continuously engaged in HIV care until the point of ART eligibility [17]. Other reviews of losses along the HIV care continuum in sub-Saharan Africa have estimated that 54%–69% of those ineligible for ART at the time of HIV care enrollment are lost to care prior to ART initiation, with the poorest retention rates among adolescents, young adults, and those enrolling in care at earlier stages of infection [18,19]. In contrast, among patients eligible for ART at the time of enrollment into care, the proportions lost to care prior to ART initiation were 25%–36% [18,19]. In a recent analysis from South Africa, which compared outcomes among patients just below and just above the ART eligibility cutoff, patient eligibility for immediate treatment was associated with a 25 percentage point (pp) increase in ART initiation and an 18 pp increase in 12-month retention [20].

Using longitudinal data on patients enrolling in HIV care from the International Epidemiology Databases to Evaluate AIDS (IeDEA) network, we sought to assess changes in timely ART initiation at the original site of care enrollment following major expansions in HIV treatment guidelines across multiple countries and regions and to identify factors associated with these changes.

## Methods

### Data sources and management

#### Patient data

IeDEA (www.iedea.org) contains sociodemographic and clinical data for over 1.7 million patients enrolled in HIV care in 46 countries across 7 regional cohort collaborations [21]. The vast majority (87%) of IeDEA sites are public sector health facilities, including both primary-level sites (42%) and secondary/tertiary-level sites (58%) [22], thus representing a diverse population of HIV patients. Almost all IeDEA sites offer HIV counseling and testing on-site, and common entry points into HIV care at these sites are internal referrals from other units (i.e., HIV counseling and testing), antenatal care, outpatient departments, and referrals from other health facilities, as well as community-based testing programs in their catchment areas [22,23]. In this analysis, we used prospectively and retrospectively collected data from 2006–2017 for 6 regional cohorts with pre-ART data available (Asia-Pacific, Central and South America, Central Africa, East Africa, North America, and Southern Africa). Prior to data merge and analysis, each region’s data were harmonized by regional data managers in accordance with IeDEA data exchange standards regarding variable definitions, data cleaning, and formatting.

### ART guidelines

We conducted a systematic search for current and historical ART eligibility guidelines of countries participating in IeDEA, including sources such as the Joint United Nations Programme on HIV/AIDS (UNAIDS) Database of National HIV Guidelines [24], the International Association of Providers of AIDS Care (IAPAC) Global HIV Watch [25], the HIV Treatment Guideline Database, health ministry websites, search engines, and published literature, including ART guideline reviews [26–28]. For countries with no publicly available ART guidelines, we obtained information from in-country HIV clinicians, researchers, and ministry of health officials. We identified the time points when each country expanded ART eligibility to include asymptomatic people living with HIV (PLWH) with CD4 counts ≤350 cells/μL and ≤500 cells/μL. We also identified concurrent expansions of guidelines related to expanded treatment for pregnant women and patients coinfected with tuberculosis, regardless of other eligibility criteria (i.e., CD4 counts or clinical staging). If the exact month of ART eligibility expansion was unknown, a midyear value was used.

This cohort study utilized deidentified data approved for use by local ethical committees in each of the IeDEA regions included in the analysis.

### Eligibility criteria

Sites eligible for inclusion had to have HIV care data available on patients for the period after care enrollment but preceding ART initiation (pre-ART data), as well as data on both ART initiators and noninitiators. Patients had to be at least 16 years old at enrollment (18 in North America) and have at least 12 months of possible follow-up between enrollment and database closure. Patients were excluded if they transferred to an IeDEA site from another clinic, lacked a clinic visit and lab record, or were known not to be ART naïve at enrollment (with ART defined as any regimen of at least 3 antiretroviral drugs, excluding treatment taken for prevention of maternal-to-child transmission).

To ensure that there was no differential selection into the sample by ART eligibility, distributions of CD4 counts at enrollment were assessed with respect to possible discontinuities at the point of ART eligibility (CD4 = 350 and CD4 = 500 for the 2 analyses), which would indicate potential exclusion of non-ART-eligible patients from contributing sites’ cohorts (S1 Text).

### Outcome

The outcome of interest was site-level absolute pp change in cumulative incidence of ART initiation (CI-ART) within 6 months of enrollment at each site—namely, the difference in CI-ART between those enrolling in HIV care 6–18 months prior to national ART eligibility expansion (“pre” or baseline period) and those enrolling 6–18 months thereafter (“post” period). Pp changes were computed separately for ART eligibility expansions to CD4 counts ≤350 cells/μL and ≤500 cells/μL. Patients enrolling in the 6 months immediately preceding and following guideline introduction were excluded to allow for early or lagged implementation of national guidelines at the site level and to ensure that the 6-month CI-ART outcome for the “pre” period occurred before the guideline change. Additionally, to ensure sufficient data for reliable outcome estimation, sites that had fewer than 30 study eligible patients who enrolled in either of the “pre” and “post” periods bracketing ART eligibility expansion were excluded.

To derive the outcome measure, we estimated overall 6-month CI-ART separately for the “pre” and “post” ART eligibility expansion periods via competing risks regression with the Aalen-Johansen estimator, treating death and pre-ART loss to clinic as competing events. To minimize the risk of misclassifying temporarily disengaged patients as lost, pre-ART loss to clinic was defined as not returning to clinic for at least 12 months, with no evidence of subsequent return.

### Statistical and analytic methods

The CI-ART was estimated via competing risk methods, with the Aalen-Johansen estimator, and treating death and loss to clinic as competing risks, as outlined in a July 2016 concept proposal to the IeDEA Executive Committee (S2 Text), which also prespecified inclusion and exclusion criteria, outcome definitions, and key stratification variables. As discussed in the appendix (S2 Text), other analytic approaches, including the use of site-level metaregression and the pre-post within-site comparisons to derive the outcome, were selected after data became available.

To assess variability of change in 6-month CI-ART across sites, forest plots were used to visualize site-level outcomes, and between-site variances (tau-squared) and *p*-values from chi-squared tests of between-site heterogeneity were calculated.

### Pooled estimates and stratification

We calculated pooled summary estimates of the pp change in 6-month CI-ART across all sites for each major expansion in treatment eligibility criteria, along with corresponding 95% confidence intervals (CIs). We stratified all estimates across several site and patient characteristics. Site characteristics included IeDEA region (see “Data Sources”), site setting (urban versus rural), and site-level summary measures of the patient population enrolling at the site, including overall proportion of female clients (≤45%, 45%–65%, >65%); baseline 6-month CI-ART (grouped in quartiles) among all patients and among those previously eligible for treatment as a site-level measure of the preexisting service level and capacity to expand; and proportion of patients eligible for treatment in the “post” period, under expanded guidelines, as a measure of unmet need for treatment. Patient characteristics included sex, age at enrollment (dichotomized into 16–24 years and ≥25 years, to align with the 15–24 age category that is commonly used by WHO [29]), and CD4 count at enrollment. CD4 count at enrollment was categorized to reflect patients’ ART eligibility status vis-à-vis each guideline change: newly eligible (CD4 count 201–350) and previously eligible (CD4 ≤ 200) for the ART eligibility expansion to CD4 ≤ 350 cells/μL and newly eligible (CD4 351–500) and previously eligible (CD4 ≤ 350), in analyses of ART eligibility expansion to CD4 ≤ 500 cells/μL.

### Metaregression models

Linear random effects metaregression models were used to examine factors associated with changes in 6-month CI-ART while controlling for concurrent expansions of other ART eligibility criteria and to assess the predicted magnitude of change in 6-month CI-ART following ART eligibility expansions. Bivariate and multivariable associations between the pp change in 6-month CI-ART and site and patient population characteristics were examined through linear random effects metaregression, adjusted for concurrent expansions of ART eligibility criteria for pregnant women and tuberculosis patients. To meet the assumption of linearity in the association between continuous independent variables and the outcome, cohort size was log transformed.

Covariates used in metaregression models included variables related to site characteristics (i.e., baseline 6-month CI-ART, baseline median enrollment CD4, median age at enrollment, and proportion of female patients at a site), as well as binary variables related to concurrent guideline changes extending HIV treatment to all pregnant women and tuberculosis patients. Site-level covariates were derived separately for each of the 2 ART eligibility expansions (to CD4 ≤ 350 cells/μL and to CD4 ≤ 500 cells/μL), based on each site’s patient population in the periods bracketing each ART eligibility expansion.

Analyses were completed in Stata 14.

## Results

Overall, 260 IeDEA sites had provided longitudinal data on at least 60 patients—the minimum for potential inclusion in the analysis. Thirty sites were further excluded because of lack of data on pre-ART patients. Among the remaining 230 sites, 225 were in countries where eligibility expansions of interest occurred during the period under study. Among these sites, 171 had sufficiently large cohorts for inclusion in this analysis (i.e., at least 30 patients meeting the eligibility criteria described above in each 12-month period bracketing guideline expansion).

### ART eligibility expansions and site characteristics

The analysis of ART eligibility expansion to treat asymptomatic PLWH with CD4 count ≤350 cells/μL included 145 sites (with 169,717 patients) in 22 countries where guidelines changed between 2007 and 2012. Six of these countries revised national treatment eligibility guidelines before WHO’s November 2009 recommendation [30] to initiate treatment at CD4 ≤ 350, by a mean of 15 months (range 5–27 months). Among the 16 countries that adopted expanded guidelines after WHO’s recommendation, the mean time elapsed was 20 months (range 6–31 months).

The analysis of ART eligibility expansion for asymptomatic PLWH with CD4 count ≤500 cells/μL included 152 sites (with 128,552 patients) in 15 countries where guidelines changed between 2009 and 2015. Three of these countries introduced new guidelines prior to WHO’s June 2013 recommendation [31] on treatment eligibility (mean of 28 months; range 11–42 months). Among the remaining 12 countries, national guideline change followed WHO recommendations by a mean of 9 months (range 1–24 months).

For both analyses, half of the sites were from Southern Africa (50% and 51%, respectively; Table 1).

**Table 1:** Characteristics of 171 sites and 284,740 patients included in the analyses.

			Guideline expansion to CD4 ≤ 350			Guideline expansion to CD4 ≤ 500			Sites^a^ included in both analyses
	Overall		Sites	Patients		Sites	Patients		
	*N* countries	*N* sites	*N* %	*N*	%	*N* %	*N*	%	*N* (%)
**Overall**	22	171	145 100%	169,717	100%	152 100%	128,552	100%	126/171 (74%)
**Region**									
** Asia-Pacific**	Cambodia, Hong Kong, India, Indonesia, and Vietnam	6	6 4%	2,868	2%	2 1%	378	<1%	2/6 (33%)
** Central Africa**	Burundi, DRC, and Rwanda	14	12 8%	5,886	3%	13 9%	3,559	3%	11/14 (79%)
** East Africa**	Kenya, Tanzania, Uganda	50	36 25%	42,804	25%	46 30%	36,856	29%	32/50 (64%)
** North America**	Canada and United States	13	13 9%	7,262	4%	10 7%	6,949	5%	10/13 (77%)
** Southern Africa**	Malawi, South Africa, Zambia, and Zimbabwe	83	73 50%	109,012	64%	78 51%	79,664	62%	68/83 (82%)
** South and Central America**	Argentina, Brazil, Chile, Mexico, and Peru	5	5 3%	1,885	1%	3 2%	1,146	1%	3/5 (60%)
**Site setting**									
** Urban**		75	72 50%	81,077	48%	65 43%	60,410	47%	62/75 (83%)
** Rural**		57	42 29%	61,200	36%	52 34%	45,690	36%	37/57 (65%)
** Unknown**		39	31 21%	27,440	16%	35 23%	22,452	17%	27/39 (69%)
**Overall cohort characteristics (“pre” and “post” groups)**									
** Median age at enrollment (IQR)**			34 (32–35)			33 (32–35)			
** Median % women in cohort (IQR)**			62 (60–66)			63 (59–66)			
**Cohort characteristics at baseline (“pre” group)**									
** Median % with CD4 at enrollment (IQR)**			86 (77–92)			67 (53–80)			
** Median CD4 at enrollment (IQR)**			223 (198–290)			273 (243–331)			
** Median % newly ART eligible under expanded guidelines (IQR)**^**b**^^,^^**c**^			19 (16–23)			12 (8–15)			
**Cohort characteristics after expansion (“post” group)**									
** Median % with CD4 at enrollment (IQR)**			80 (64–88)			61 (37–79)			
** Median CD4 at enrollment (IQR)**			261 (230–296)			284 (248–344)			
** Median % newly ART eligible under expanded guidelines (IQR)**^**b**^^,^^**c**^			19 (15–21)			11 (8–15)			

Abbreviations: ART, antiretroviral treatment; DRC, Democratic Republic of the Congo.

^a^ There were 13,529 patients included in both analyses (in the “post” period of the expansion to CD4 ≤ 350 analysis and the “pre” period in the expansion to CD4≤500 analysis)

^b^ Those with unknown eligibility were included in the denominator

^c^ Defined as patients with an enrollment CD4 count between 201 and ≤350 (in the expansion to CD4 ≤ 350 analysis) or 351 and ≤500 (in the expansion to CD4 ≤ 500 analysis).

During the periods preceding ART eligibility expansions to CD4 ≤ 350 and CD4 ≤ 500, the median proportion of patients initiating ART within 6 months of enrollment was 53.3% and 57.5% of patients, respectively (Table 2).

**Table 2:** Overall and pooled change in 6-month cumulative incidence of antiretroviral treatment initiation (CI-ART) after ART eligibility guideline expansion to CD4 ≤ 350 and CD4 ≤ 500.

	Guideline expansion to CD4 ≤ 350				Guideline expansion to CD4 ≤ 500			
	*N* sites	Median baseline 6-month CI-ART (IQR)	*N* patients	Estimate (95% CI)	*N* sites	Median baseline 6-month CI-ART (IQR)	*N* patients	Estimate (95% CI)
**Overall**	145	53 (46–59)	169,717	4.3 (2.6–6.1)	152	57 (50–64)	128,552	15.9 (14.3–17.4)
**Patient level (medians and estimates of change in 6-month CI-ART are restricted to patient strata)**								
**Sex**^**a**^								
** Men**	129	56 (48–64)	66,656	4.1 (2.2–5.9)	117	61 (52–68)	50,116	13.7 (12.0–15.4)
** Women**	127	54 (45–58)	101,284	4.6 (2.6–6.5)	131	58 (50–64)	74,154	16.8 (15.0–18.6)
**Age at enrollment**^**a**^								
** 16–24 years**	90	45 (35–51)	28,150	4.4 (2.0–6.8)	82	51 (45–58)	22,210	21.5 (18.9–24.2)
** ≥25 years**	143	55 (48–62)	139,228	4.4 (2.6–6.1)	148	60 (50–67)	102,974	15.5 (13.8–17.3)
**CD4 count at enrollment**^**b**^								
** CD4 201–350 (newly eligible)**	91	61 (50–70)	29,964	18.2 (15.3–21.1)				
** CD4 ≤ 200 (previously eligible)**	91	84 (80–88)	49,615	−0.6 (−2.0–0.7)				
** CD4 351–500 (newly eligible)**					52	36 (27–45)	10,824	47.4 (41.5–53.4)
** CD4 ≤ 350 (previously eligible)**					52	84 (78–88)	36,199	4.9 (3.3–6.5)
**Site level (medians and estimates of change in 6-month CI-ART are for overall site population)**								
**Region**								
** Asia-Pacific**	6	74 (47–76)	2,868	12.0 (-4.2–28.1)	2	73 (62–83)	378	13.5 (-0.5–27.4)
** Central Africa**	12	37 (31–46)	5,886	7.3 (-3.5–18.1)	13	55 (52–61)	3,559	7.4 (0.4–14.3)
** East Africa**	36	53 (47–56)	42,804	7.6 (4.3–10.9)	46	53 (47–59)	36,856	15.8 (12.9–18.6)
** North America**	13	46 (40–55)	7,262	4.6 (-0.2–9.4)	10	50 (43–59)	6,949	13.7 (9.4–18.1)
** Southern Africa**	73	56 (49–62)	109,012	1.7 (-0.5–3.8)	78	60 (51–66)	79,664	17.6 (15.2–20.0)
** South and Central America**	5	54 (52–68)	1,885	4.9 (-4.1–13.7)	3	62 (61–71)	1,146	12.1 (6.5–17.7)
**Site setting**								
** Urban**	72	52 (41–58)	81,077	5.3 (3.0–7.7)	65	58 (52–62)	60,410	14.2 (12.1–16.3)
** Rural**	42	56 (50–60)	61,200	4.7 (1.3–8.1)	52	56 (48–66)	45,690	16.7 (14.0–19.4)
** Unknown**	31	52 (42–62)	27,440	1.4 (-2.4–5.3)	35	61 (50–65)	22,452	17.7 (12.7–22.7)
**% female (overall, in “pre” and “post” periods)**								
** ≤45%**	24	53 (41–63)	12,006	6.5 (1.4–11.6)	17	60 (46–62)	9,142	14.7 (11.4–18.1)
** >45–65%**	75	53 (46–60)	109,189	3.8 (1.4–6.2)	88	60 (49–66)	92,559	17.1 (14.9–19.4)
** >65%**	46	54 (45–58)	48,522	4.4 (1.5–7.2)	47	53 (50–59)	26,851	13.8 (11.0–16.5)
**Baseline (“pre” period) 6-month CI-ART among all patients**								
** Top quartile**	37	66 (62–71)	56,226	0.4 (−2.2–2.9)	38	69 (66–74)	48,519	10.6 (8.6–12.7)
** Middle-high quartile**	36	56 (54–58)	53,729	3.0 (−0.1–6.0)	38	61 (59–62)	35,632	16.8 (14.4–19.2)
** Middle-low quartile**	35	50 (47–52)	30,726	3.0 (−0.2–6.1)	38	53 (52–55)	26,486	12.4 (9.6–15.1)
** Bottom quartile**	37	37 (32–41)	29,036	11.8 (7.4–16.2)	38	42 (38–47)	17,915	26.2 (20.7–31.7)
**Baseline (“pre” period) 6-month CI-ART among previously ART eligible patients**^**c**^								
** Top quartile**	32	57 (48–67)	21,874	−0.1 (−3.5–3.4)	32	63 (60–69)	23,656	12.9 (10.2–15.7)
** Middle-high quartile**	32	56 (52–62)	35,844	3.7 (−0.1–7.5)	33	63 (58–68)	38,794	14.4 (12.0–16.9)
** Middle-low quartile**	32	55 (49–60)	60,833	5.9 (2.2–9.7)	31	57 (51–62)	35,175	16.2 (12.8–19.7)
** Bottom quartile**	32	51 (40–55)	48,158	5.5 (2.8–8.2)	33	47 (42–52)	26,946	19.3 (13.8–24.7)
**“post” period % ART eligible under expanded guidelines**^**d**^^,^^**e**^								
** Top quartile**	36	55 (47–60)	45,601	4.9 (2.4–7.5)	37	60 (50–63)	27,394	14.6 (11.2–18.0)
** Middle-high quartile**	36	55 (45–58)	53,044	7.9 (4.4–11.4)	37	57 (49–63)	32,586	16.9 (13.9–20.0)
** Middle-low quartile**	36	55 (49–61)	37,526	2.6 (−0.2–5.5)	37	59 (52–65)	37,936	16.0 (13.0–19.0)
** Bottom quartile**	37	47 (39–54)	33,546	1.4 (−3.2–5.9)	37	55 (48–62)	29,114	16.2 (11.7–20.8)

^a^ Includes only sites with ≥30 patients in each “pre” and “post” period in the given stratum

^b^ Includes only sites with ≥30 patients in each “pre” and “post” period in both strata

^c^ Includes only sites with ≥30 previously eligible patients in the “pre” period

^d^ Includes only sites with ≥10 patients with known ART eligibility in the “post” period

^e^ Patients with unknown eligibility included in denominator of proportions.

Patient demographics were similar across sites, with a median age of 34 years and female patients comprising more than 60% of patients on average. Baseline median CD4 counts at enrollment into HIV care were higher among sites included in the analysis of CD4≤500 ART eligibility expansion than among the CD4≤350 ART eligibility expansion sites (273 cells/μL versus 223 cells/μL). However, a larger proportion of patients were missing data for CD4 count at enrollment during both the “pre” and “post” periods in the analysis of the second ART eligibility expansion (median 33% versus 14% and 39% versus 20%, respectively; Table 1).

### Pooled estimates of change in 6-month CI-ART following ART eligibility expansions

**Fig 1:**
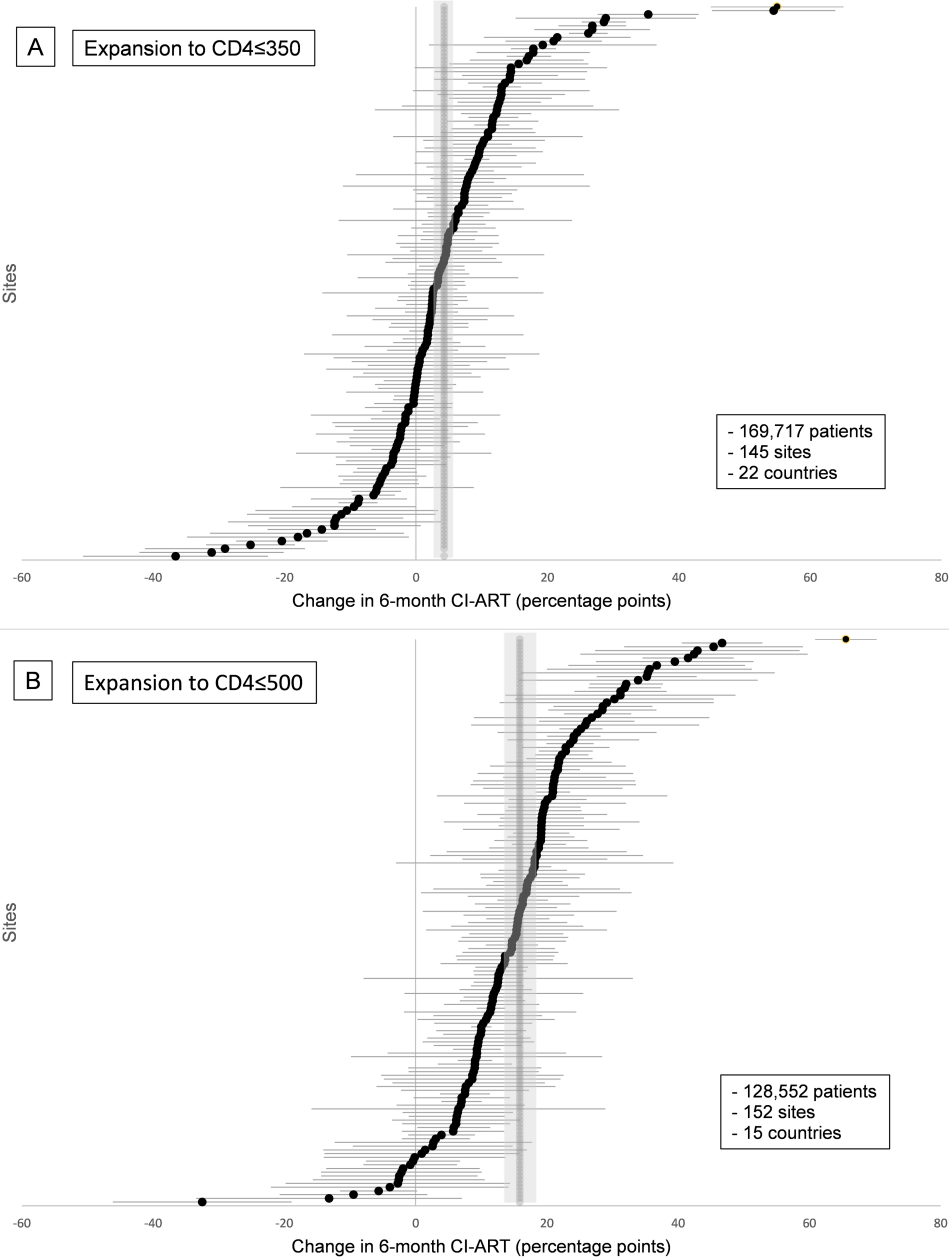
Site-level changes in 6-month cumulative incidence of antiretroviral treatment initiation (CI-ART) after guideline expansions to (A) CD4 ≤ 350 and (B) CD4 ≤ 500. Pooled estimates and confidence intervals are in gray.

Marked changes in CI-ART were observed after each expansion in treatment eligibility: 4.3 pp (95% CI 2.6–6.1 pp) after the expansion to CD4 ≤ 350 cells/μL and 15.9 pp (95% CI 14.3–17.4 pp) after the expansion to CD4 ≤ 500 cells/μL (Table 2). There was considerable heterogeneity in site-level changes in CI-ART for both guideline expansions, and heterogeneity chi-squared tests were statistically significant (both *p* <0.001). However, a much larger proportion of sites experienced positive changes in 6-month CI-ART following the latter ART eligibility expansion to CD4 ≤ 500 cells/μL (Fig 1A and 1B). Overall, 33% of sites (48/145) had statistically significant increases in 6-month CI-ART after the expansion to CD4 ≤ 350 cells/μL, 57% of sites (83/145) had no statistically discernible change, and 10% (14/145) had statistically significant decreases. After expansion to CD4 ≤ 500 cells/μL, 74% of sites (112/152) had statistically significant increases, 26% of sites (39/152) had no statistically discernible change, and <1% (1/152) had a statistically significant decrease.

In stratified analyses of the guideline expansion to treat patients with CD4 ≤ 350 cells/μL, changes in 6-month CI-ART were most pronounced at sites in Asia-Pacific, East Africa, and Central Africa (12.0, 7.6 and 7.3 pp, respectively), at sites with higher proportions of newly eligible patients in the “post” period (4.9 pp and 7.9 pp for clinics in the top 2 quartiles of the proportion of patients newly eligible for treatment, respectively), and sites in the lowest quartile of “pre” period 6-month CI-ART (11.8 pp). There was also a trend in the association between lower “pre” period 6-month CI-ART among previously eligible patients specifically and greater site-level change in CI-ART after guideline expansion (Table 2).

Following the CD4 ≤ 500 expansion, the largest changes in CI-ART were observed in Southern and East Africa (17.6 pp and 15.8 pp, respectively). As observed in the CD4 ≤ 350 analysis, there was a trend of greater change in 6-month CI-ART with lower “pre” period 6-month CI-ART (overall and among previously eligible patients specifically); sites in the lowest quartile of “pre” period 6-month CI-ART had the largest changes in CI-ART following ART eligibility expansion (26.2 pp). However, in contrast to trends observed following expansion to CD4 ≤ 350, there was no trend in CI-ART across quartiles of proportion of newly eligible patients in the “post” period (Table 2).

**Fig 2:**
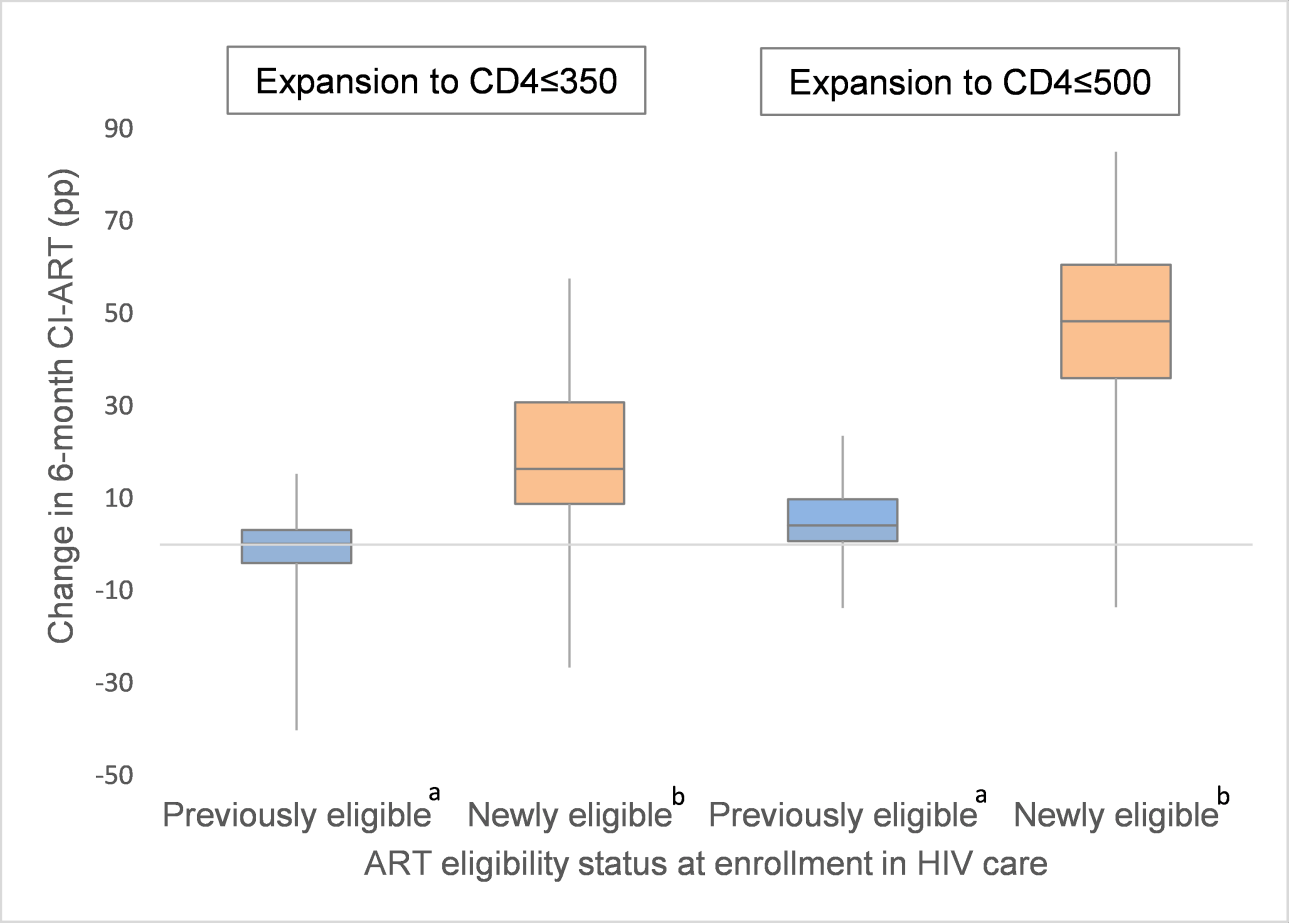
Distribution of site-level changes in 6-month cumulative incidence of antiretroviral treatment initiation (CI-ART) after expansions to CD4 ≤ 350 and CD4 ≤ 500, by patient ART eligibility status at enrollment. ^a^ Defined as those enrolling at CD4 ≤ 200 (in the expansion to CD4 ≤ 350 analysis) and those enrolling at CD4 between 201 and 350 (in the expansion to CD4 ≤ 500 analysis). ^b^ Defined as those enrolling at CD4 ≤ 350 (in the expansion to CD4 ≤ 350 analysis) and those enrolling at CD4 between 351 and 500 (in the expansion to CD4 ≤ 500 analysis).

In analyses stratified by patient characteristics, no significant differences in the pp change in CI-ART were observed by sex or age after expansion to CD4 ≤ 350 cells/μL. However, after expansion to CD4 ≤ 500 cells/μL, appreciably larger increases were observed among women when compared to men (16.8 versus 13.7 pp) and among patients aged 16–24 years at enrollment, compared to those 25 years or older (21.5 versus 15.5 pp). Striking differences were also observed in pooled estimates of change in CI-ART by CD4 count at enrollment. Specifically, while no or minimal improvement was observed among patients who were already ART eligible before guideline expansion, a large increase was noted among those newly eligible for treatment under the expanded ART criteria (18.2 pp among newly eligible patients after expansion to CD4 ≤ 350 cells/μL and 47.4 pp among newly eligible patients after expansion to CD4 ≤ 500 cells/μL; Table 2, Fig 2).

### Metaregression: Predicted and adjusted effects

Six-month CI-ART during the period before ART eligibility expansion was the strongest correlate of change in CI-ART following eligibility expansion. In bivariate analyses, the predicted change in 6-month CI-ART after expansion to CD4 ≤ 350 was 9.1 pp for a site with “pre”-period CI-ART of 40% and 1.4 pp for a site with “pre”-period CI-ART of 60%. After expansion to CD4 ≤ 500, the predicted changes in CI-ART were 24.1 pp and 14.6 pp, respectively. In adjusted metaregression models, “pre”-period 6-month CI-ART was the strongest correlate of pp change in 6-month CI-ART after ART eligibility expansion (Table 3). Adjusted changes in CI-ART following both ART eligibility expansions were greatest among those sites with lower pre-expansion levels of CI-ART; each 10-unit decrease in the “pre”-period 6-month CI-ART was associated with an average 3.9 and 5.3 pp change in CI-ART after CD4 ≤ 350 and CD4 ≤ 500 cells/μL eligibility expansions, respectively. Increasing cohort size was also positively associated with change in CI-ART (Table 3).

**Table 3:** Crude and adjusted metaregression analyses of change in 6-month cumulative incidence of antiretroviral treatment initiation (CI-ART) after ART eligibility guideline expansion to CD4 ≤ 350 and CD4 ≤ 500.

		Guideline expansion to CD4 ≤ 350^a^		Guideline expansion to CD4 ≤ 500^a^	
Site characteristic	Unit	Crude Beta (95% CI)	Adjusted^a^ Beta (95% CI)	Crude Beta (95% CI)	Adjusted^a^ Beta (95% CI)
**Baseline 6-month CI-ART**	10 pp decrease	**3.8 (2.3–5.3)**	**3.9 (2.4–5.4)**	**4.8 (3.4–6.1)**	**5.3 (3.9–6.7)**
**Baseline median CD4 at enrollment**	10 cell/μl increase	**0.4 (0.2–0.7)**		0.0 (−0.3–0.3)	
**Median age at enrollment**	1-year increase	−0.5 (−1.1–0.2)		0.0 (−0.6–0.6)	
**Proportion female**	1-pp increase	−0.1 (−0.2–0.1)		0.0 (−0.1–0.1)	
**Cohort size**	1-log increase	1.0 (−1.0–3.0)	**2.6 (0.8–4.5)**	0.0 (−1.9–1.9)	**2.0 (0.3–3.7)**
**Setting**	Urban	0.6 (−4.0–5.3)		−2.7 (−7.0–1.6)	
	Rural	Reference		Reference	
	Unknown	−3.3 (−9.0–2.4)		1.1 (−3.9–6.2)	

^a^ The model was also adjusted for ART eligibility concurrently expanded to encompass all individuals with tuberculosis or pregnancy.

Metaregression models adjusting for site, patient population, and guideline characteristics explained 24.1% and 38.1% of between-site variability, with tau-squared reduced from 126.1 to 95.7 and from 110.4 to 68.3 in the CD4 ≤ 350 and CD4 ≤ 500 analyses, respectively.

## Discussion

Our study found that expansions in ART eligibility criteria for people with HIV infection were followed by substantial increases in rates of timely ART initiation at the original clinic of enrollment, with much larger increases among those newly eligible under the expanded guidelines compared with those eligible under prior guidelines. Importantly, with expansion to CD4 ≤ 500, increases were most pronounced for persons aged 16–24 years, a critical population that has historically been particularly difficult to engage in care. For both ART eligibility expansions examined (i.e., to CD4 ≤ 350 and CD4 ≤ 500), changes in 6-month CI-ART were greatest at sites that had lower rates of ART initiation prior to ART eligibility expansion and, in metaregression analyses, “pre”-period rates of ART initiation were significantly and inversely associated with changes in CI-ART following guideline change. Larger increases in CI-ART overall were observed following the adoption of CD4 count ≤500 cells/μL treatment eligibility guidelines, with statistically significant increases in CI-ART observed at 74% of sites and a dramatic shift in the pooled estimate of CI-ART across all sites included in the analysis.

Concern has been expressed that expanding ART eligibility may result in sick patients being “crowded out” by asymptomatic patients [32]. Importantly, and consistent with research from South Africa [33,34], increases in ART initiation among newly eligible patients on average were not offset by decreases in ART initiation rates among previously eligible patients, meaning that access to treatment by sicker patients with lower CD4 counts did not decrease when patients with higher CD4 counts became eligible and started treatment. In fact, sites with the lowest baseline ART initiation rates among previously eligible patients saw the greatest overall increases in ART initiation after guideline expansion, which also suggests that broadened treatment eligibility did not compromise access for sicker patients.

Another major finding of our study is that increases in timely ART initiation were largest among young adults—a group that has been consistently shown to have lower rates of retention in care both prior to and after ART initiation, as well as lower rates of ART initiation at early stages of infection [35–38]. Improving timely ART initiation among young adults is particularly important, given that this age group accounts for almost half of new HIV infections among adults [39].

To our knowledge, no analyses to date have assessed differences in ART initiation following major changes in HIV treatment eligibility criteria across multiple countries and regions. While the results of this analysis are promising, further research is needed to assess how increases in ART initiation at higher CD4 counts and among asymptomatic patients affect retention in the HIV care continuum over the longer term, including ART adherence, and sustained HIV virologic suppression. While some studies have suggested increased loss to follow-up on ART among patients with higher CD4 counts [40,41], evidence increasingly supports improved retention among patients initiating with higher CD4 counts [15], including those immediately eligible for ART versus those just under the eligibility threshold [20], and same-day (or “rapid”) ART initiators [42], with any increases in loss to follow-up more than offset by eliminating opportunity for pre-ART attrition [43].

The results of this study are important, given a recent systematic review of national HIV treatment cascades, which indicated that the most common “break point” in the HIV care cascade is between diagnosis and initiation of ART [10], which is critical for achieving optimal HIV care outcomes at the individual and population level [44]. In conjunction with efforts to improve HIV testing and linkage to care [45,46], in part via point-of-care CD4 testing to assess patients’ ART eligibility [47], accelerating the adoption of expanded treatment eligibility criteria, including WHO’s current “Treat All” recommendation, could help reduce rates of pre-ART loss to clinic and increase rates of timely ART initiation. Mathematical models suggest that improving rates of HIV testing, linkage, and immediate ART initiation, combined with other prevention interventions, could nearly stop HIV transmission [48].

At the country level, however, treatment guidelines often lag behind WHO guidance, creating missed opportunities to realize the preventive potential of ART [26,27]. A recent analysis of HIV treatment policy changes in sub-Saharan Africa found that the average time for the adoption of the 2013 guidelines was 10 months (range 0 to 36 months) [26]. Compounding delays in the national adoption of global guidance are lags in the practical implementation of new treatment policies at the service delivery level, where outdated practices among frontline health workers, treatment readiness requirements, and resource constraints lead to further delays in ART initiation among eligible HIV patients [49]. Confirming the importance of service delivery factors in promoting more timely ART initiation, a large intervention study in Uganda recently demonstrated a substantial increase in the proportion of eligible patients initiating ART within 14 days of eligibility determination from 38% to 80% (a 42 pp increase) through relatively simple and low-cost changes at the health facility level [50]. Our study results highlight the importance of promoting ART eligibility expansions, including universal test-and-treat guideline scenarios, with complementary strategies at the facility level to maximize timely diagnosis and ART initiation rates among ART-eligible persons.

Major strengths of this study include the analysis of data from a large number of patients and sites across several world regions. Patient- and site-level characteristics were used to provide setting-specific estimates and to adjust for some differences in patient mix across sites. Where known, concurrent expansions of ART eligibility criteria regarding pregnancy and tuberculosis status were also controlled for, to account for varied scopes and foci of guideline changes. Findings from this large and heterogeneous sample of real-world service delivery settings may be generalizable to many contexts, especially where substantial room remains to expand ART eligibility guidelines. Additionally, the use of a 12-month buffer period around the national guideline change date helped both to reduce exposure misclassification and to control for differences in patient mix and seasonal patterns of ART initiation in within-site pre-post comparisons.

However, even with the use of a buffer period, the lack of data on the exact timing of site-level implementation of expanded ART eligibility guidelines likely contributed to imprecision of the site-level CI-ART estimates, as implementation of expanded guidelines may have preceded national adoption at some sites and lagged at other sites. Furthermore, the inclusion of 171 sites from 22 countries necessarily means considerable heterogeneity of ART referral and record-keeping practices, which may have also affected the precision of the estimates. With half of all sites and over 60% of patients in this analysis being from Southern Africa, overall estimates are strongly influenced by this region. However, changes in CI-ART were similar across regions, especially in the expansion to CD4≤500 analysis, albeit with some regions having lower volumes of data (Asia-Pacific and South and Central America) and therefore less precise CI-ART estimates.

While many sites experienced increases in timely ART initiation, particularly after treatment eligibility was expanded to CD4 ≤ 500 cells/μL, no change in CI-ART and occasional decreases in CI-ART were also observed, possibly as a result of unmeasured site-level factors (e.g., medication or reagent shortages, staffing changes, and other resource constraints). We did not have data on many facility- and patient population-related factors that could help illuminate the reasons behind observed decreases in ART initiation at selected sites after country-level guideline expansion. In addition, random fluctuation or regression to the mean may help explain some decreases and increases (for example, changes observed among sites with lowest “pre”-period ART initiation rates).

Nuanced expansions in national ART eligibility criteria related to clinical stage and pregnancy and tuberculosis status, as well as special populations, such as sex workers and serodiscordant couples, could not be reflected in this analysis. Furthermore, assumptions of a midyear policy change for countries where the exact month of ART eligibility expansion was unknown, in combination with possible early or delayed site-level implementation of national guidelines, may have obscured the effects of guideline change at the site level.

The increases in CI-ART that we observed may also overestimate the true impact of ART eligibility expansions, given our inability to directly account for secular trends in CI-ART and lack of a counterfactual within countries during the periods bracketing ART eligibility expansions. However, the change in timely ART initiation among those previously eligible after the guideline expansion is likely a good proxy for what would have happened to those newly eligible under the guideline expansion had the guideline not changed. The larger increase in timely ART initiation among those newly eligible (18.2 pp for CD4 ≤ 350 and 47.4 pp for CD4 ≤ 500) versus those previously eligible (no change for CD4 ≤ 350 and a small increase of 4.9 pp for CD4 ≤ 500) is more consistent with a guideline effect than a secular trend.

While our study design precludes causal attribution, recent research in South Africa, including studies that used regression discontinuity analysis to account for secular trends prior to and following eligibility expansions [33,45], as well as to examine ART initiation rates just above and just under eligibility threshold [20], lend support for a causal association between ART eligibility expansions and increases in ART initiation.

## Conclusions

Our study of a global sample of HIV clinics indicates that ART eligibility expansion was followed by substantial increases in timely ART initiation at the original site of enrollment, including among young adults aged 16–24 years, as well as at clinics with lower rates of timely ART initiation prior to treatment eligibility expansions. That many clinics can support the initiation of ART among newly eligible patients with less advanced disease without an adverse impact on ART initiation rates among those with more advanced disease may reflect a transition away from the emergency treatment era in some locations. While we did not have sufficient data available to assess the changes in CI-ART following the implementation of the currently recommended Treat All approach, these findings underscore the utility of ART eligibility expansion as an essential strategy in support of the UNAIDS 90-90-90 targets globally. As countries increasingly adopt WHO’s Treat All recommendation, future research should explore its impact on timely ART initiation, as well as other longer-term HIV care outcomes, including retention, ART adherence, and sustained virologic suppression.

### Disclaimer

The content of this publication is solely the responsibility of the authors and does not necessarily represent the official views of any of the governments or institutions mentioned above.

## Supporting information

S1 Checklist Strengthening the reporting of observational studies in epidemiology (STROBE) checklist for cohort studies. (DOC)

S1 Text Assessment of possible differential selection into the sample by antiretroviral treatment (ART) eligibility. (DOCX)

S2 Text Analysis concept sheet. (DOCX)

S3 Text Complete acknowledgments. (DOCX)

## Abbreviations

ART: antiretroviral treatment
CI: confidence interval
CI-ART: cumulative incidence of ART initiation
IAPAC: International Association of Providers of AIDS Care
IeDEA: International Epidemiology Databases to Evaluate AIDS
PLWH: people living with HIV
pp: percentage point(s)
UNAIDS: Joint United Nations Programme on HIV/AIDS
WHO: World Health Organization
