# Supplementary material for: HIV treatment eligibility expansion and timely antiretroviral treatment initiation following enrollment in HIV care: A metaregression analysis of programmatic data from 22 countries
Source: PLoS Med. 2018 Mar 23;15(3):e1002534. doi: 10.1371/journal.pmed.1002534 (PMC5865713; doi:10.1371/journal.pmed.1002534)
Supplement: S1 Checklist — (DOC) [file pmed.1002534.s001.doc]

STROBE Statement—C***ohort studies***

|  | Item No | Recommendation |
| --- | --- | --- |
| **Title and abstract** | 1 | (*a*) Indicate the study’s design with a commonly used term in the title or the abstract  *Title: “…meta-regression analysis…”*  *Abstract: “Random effects meta-regression models (…)”* |
|  |  | (*b*) Provide in the abstract an informative and balanced summary of what was done and what was found  *See text of the abstract.* |
| Introduction | | |
| Background/rationale | 2 | Explain the scientific background and rationale for the investigation being reported  *See paragraph 1 of Introduction.* |
| Objectives | 3 | State specific objectives, including any prespecified hypotheses  *Paragraph 3 of Introduction: “(…) we sought to assess changes in timely ART initiation following major expansions in HIV treatment guidelines across multiple countries and regions, and to identify factors associated with these changes.”* |
| Methods | | |
| Study design | 4 | Present key elements of study design early in the paper  *Paragraph 1 of Methods: “(…) prospectively and retrospectively collected data (…) for six regional cohorts (…)”* |
| Setting | 5 | Describe the setting, locations, and relevant dates, including periods of recruitment, exposure, follow-up, and data collection  *Paragraph 1 of Methods: “(…) data from 2006-2017 for six regional cohorts with pre-ART data available (Asia-Pacific; Central and South America; Central Africa; East Africa; North America; and Southern Africa)”* |
| Participants | 6 | (*a*) Give the eligibility criteria, and the sources and methods of selection of participants. Describe methods of follow-up  *See paragraphs 4- 6 of Methods.* |
|  |  | (*b*) For matched studies, give matching criteria and number of exposed and unexposed  *N/A* |
| Variables | 7 | Clearly define all outcomes, exposures, predictors, potential confounders, and effect modifiers. Give diagnostic criteria, if applicable  *Outcome: See paragraphs 6 & 7 of Methods.*  *Predictors: See paragraphs 10- 12 of Methods.* |
| Data sources/ measurement | 8* | For each variable of interest, give sources of data and details of methods of assessment (measurement). Describe comparability of assessment methods if there is more than one group  *Data sources: See paragraphs 1 & 2 of Methods.*  *Measurement: See paragraphs 10 & 12 of Methods.* |
| Bias | 9 | Describe any efforts to address potential sources of bias  *See paragraphs 5 & 7 of Methods.* |
| Study size | 10 | Explain how the study size was arrived at  *See paragraph 1 of Results.* |
| Quantitative variables | 11 | Explain how quantitative variables were handled in the analyses. If applicable, describe which groupings were chosen and why  *See paragraphs 10-12 of Methods.* |
| Statistical methods | 12 | (*a*) Describe all statistical methods, including those used to control for confounding  *See paragraphs 7-12 of Methods.* |
|  |  | (*b*) Describe any methods used to examine subgroups and interactions  *See paragraph 10 of Methods.* |
|  |  | (*c*) Explain how missing data were addressed  *Tables 1, 2, and 3: Missing category for urban/rural site setting* |
|  |  | (*d*) If applicable, explain how loss to follow-up was addressed  *See paragraph 7 of Methods.* |
|  |  | (*e*) Describe any sensitivity analyses  *N/A* |
| Results | | |
| Participants | 13* | (a) Report numbers of individuals at each stage of study—eg numbers potentially eligible, examined for eligibility, confirmed eligible, included in the study, completing follow-up, and analysed  *See paragraph 1 of Results.* |
|  |  | (b) Give reasons for non-participation at each stage  *See paragraph 1 of Results.* |
|  |  | (c) Consider use of a flow diagram  *N/A* |
| Descriptive data | 14* | (a) Give characteristics of study participants (eg demographic, clinical, social) and information on exposures and potential confounders  *See paragraph 6 of Results.* |
|  |  | (b) Indicate number of participants with missing data for each variable of interest  *See table 1, paragraph 6 of Results.* |
|  |  | (c) Summarise follow-up time (eg, average and total amount)  *N/A* |
| Outcome data | 15* | Report numbers of outcome events or summary measures over time  *See paragraphs 5 & 7 of Results.* |
| Main results | 16 | (*a*) Give unadjusted estimates and, if applicable, confounder-adjusted estimates and their precision (eg, 95% confidence interval). Make clear which confounders were adjusted for and why they were included  *Unadjusted estimates: See table 3.*  *Adjusted estimates: See table 3 and paragraph 11 of Results.* |
|  |  | (*b*) Report category boundaries when continuous variables were categorized  *Results in table 2, as well as in the text of Results, report the same groupings as described in Methods* |
|  |  | (*c*) If relevant, consider translating estimates of relative risk into absolute risk for a meaningful time period  *N/A* |
| Other analyses | 17 | Report other analyses done—eg analyses of subgroups and interactions, and sensitivity analyses  *N/A* |
| Discussion | | |
| Key results | 18 | Summarise key results with reference to study objectives  *See paragraph 1 of Discussion.* |
| Limitations | 19 | Discuss limitations of the study, taking into account sources of potential bias or imprecision. Discuss both direction and magnitude of any potential bias  *See paragraphs 8-11 of Discussion.* |
| Interpretation | 20 | Give a cautious overall interpretation of results considering objectives, limitations, multiplicity of analyses, results from similar studies, and other relevant evidence  *See paragraphs 2, 3 & 4 of Discussion.* |
| Generalisability | 21 | Discuss the generalisability (external validity) of the study results  *See paragraph 7 of Discussion.* |
| Other information | | |
| Funding | 22 | Give the source of funding and the role of the funders for the present study and, if applicable, for the original study on which the present article is based  *N/A – reported in the submission system per journal’s instructions* |

*Give information separately for exposed and unexposed groups.

**Note:** An Explanation and Elaboration article discusses each checklist item and gives methodological background and published examples of transparent reporting. The STROBE checklist is best used in conjunction with this article (freely available on the Web sites of PLoS Medicine at http://www.plosmedicine.org/, Annals of Internal Medicine at http://www.annals.org/, and Epidemiology at http://www.epidem.com/). Information on the STROBE Initiative is available at http://www.strobe-statement.org.
