# Supplementary material for: HIV treatment eligibility expansion and timely antiretroviral treatment initiation following enrollment in HIV care: A metaregression analysis of programmatic data from 22 countries
Source: PLoS Med. 2018 Mar 23;15(3):e1002534. doi: 10.1371/journal.pmed.1002534 (PMC5865713; doi:10.1371/journal.pmed.1002534)

**Supplement 1: Assessment of possible differential selection into the sample by ART eligibility**

**HIV treatment eligibility expansion and timely antiretroviral treatment initiation following enrollment in HIV care – A meta-regression analysis of programmatic data from 22 countries.**

Fig A1. Distribution of CD4 counts in the period preceding guideline expansion to CD4≤350 (N=70,228).


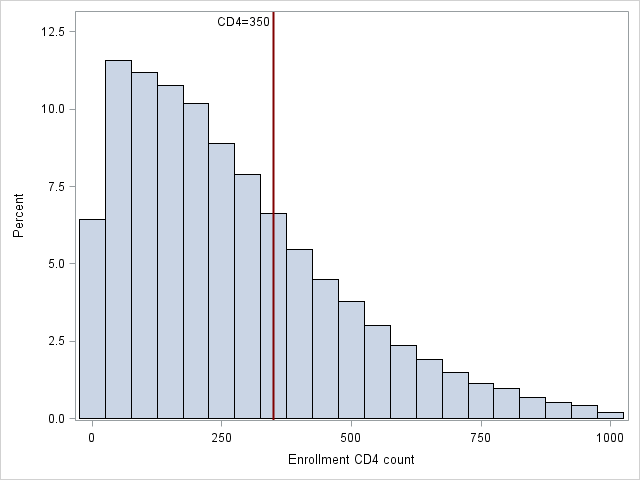


Fig A2. Distribution of CD4 counts in the period preceding guideline expansion to CD4≤350, by region.


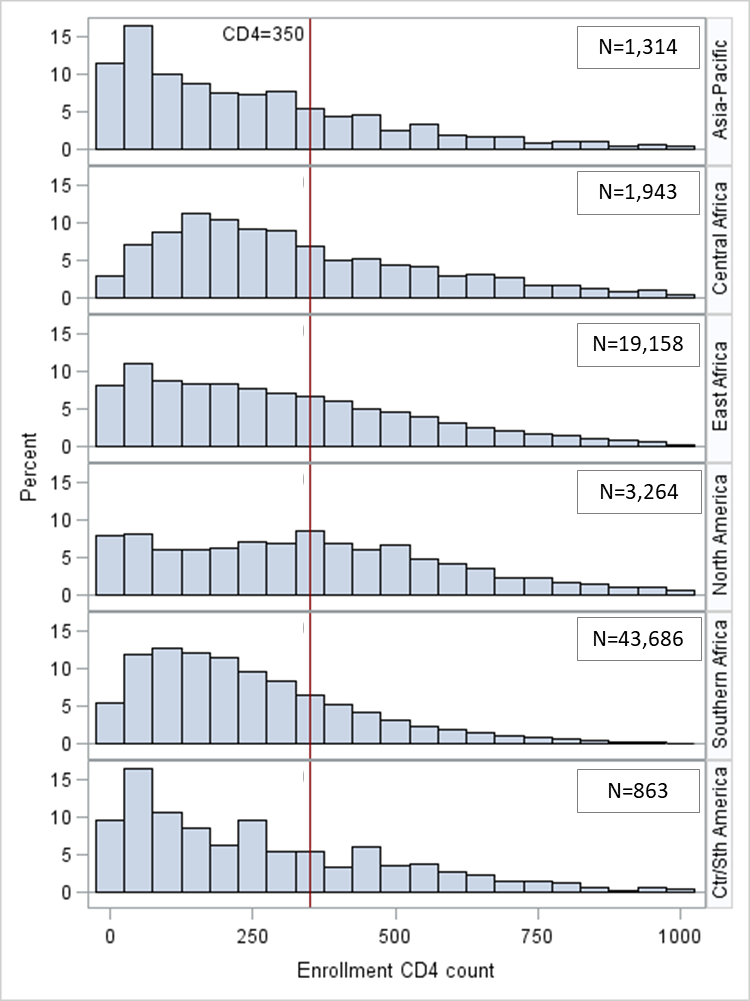


Fig A3. Distribution of CD4 counts in the period preceding guideline expansion to CD4≤500 (N=48,753).


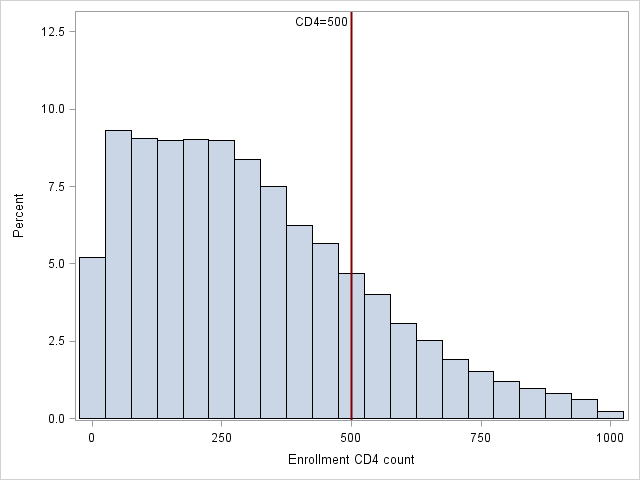


Fig A4. Distribution of CD4 counts in the period preceding guideline expansion to CD4≤500, by region.


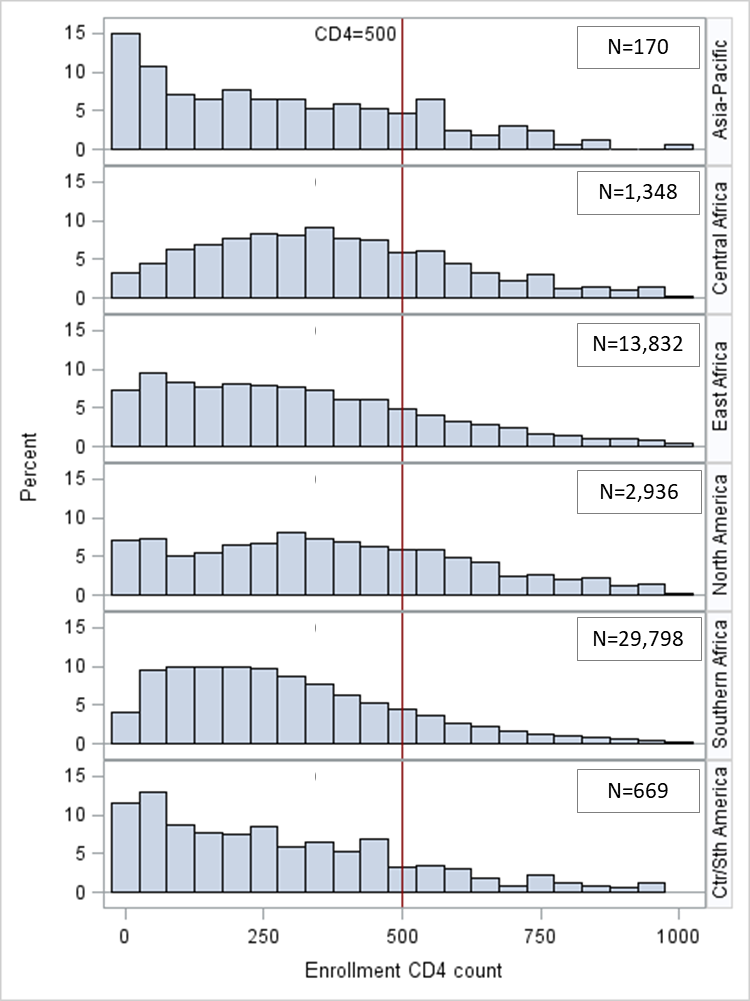

Supplement: S1 Text — (DOCX) [file pmed.1002534.s002.docx]
