# Supplementary material for: HIV treatment eligibility expansion and timely antiretroviral treatment initiation following enrollment in HIV care: A metaregression analysis of programmatic data from 22 countries
Source: PLoS Med. 2018 Mar 23;15(3):e1002534. doi: 10.1371/journal.pmed.1002534 (PMC5865713; doi:10.1371/journal.pmed.1002534)
Supplement: S2 Text — (DOCX) [file pmed.1002534.s003.docx]

**Supplement 2: Analysis concept sheet**

**HIV treatment eligibility expansion and timely antiretroviral treatment initiation following enrollment in HIV care – A meta-regression analysis of programmatic data from 22 countries.**

Olga Tymejczyk, Ellen Brazier, Constantin Yiannoutsos, Kara Wools-Kaloustian, Keri Althoff, Brenda Crabtree-Ramirez, Kinh Van Nguyen, Elizabeth Zaniewski, Francois Dabis, Jean d'Amour Sinayobye, Nanina Anderegg, Nathan Ford, Radhika Wikramanayake, and Denis Nash, for the IeDEA Collaboration

This concept sheet outlines the background, rationale, and early analytic plans for the present manuscript. It was based on a pre-existing concept outlining similar, broad analyses related to time between enrollment and ART initiation in IeDEA, which have been conducted by IeDEA annually since 2015 for an internal report to the WHO. This concept sheet was submitted to the IeDEA Executive Committee on July 29, 2016, proposing to expand selected prior analyses for journal publication.

The concept outlines the following aspects of the present analysis:

1. Estimation of cumulative incidence of ART initiation (CI-ART) via **competing risk methods, with Aalen-Johansen estimator, and treating death and loss to clinic as competing risks**
2. **Outcome** (ART eligibility, loss to clinic, ART initiation) **and other related definitions** (CD4 cell count / clinical stage at enrollment in HIV care)
3. **Key stratification variables**: sex, age, ART eligibility at enrollment in HIV care (via CD4 count and clinical stage at enrollment in HIV care), IeDEA region
4. Patient- and site-level **inclusion and exclusion criteria**
5. The use of **country-level ART eligibility expansion dates** rather than WHO recommendation dates

The key differences between this proposal and the final analysis in the manuscript are:

1. Final analysis **only includes primary objectives 3 and 4**. The remaining primary and secondary objectives were impossible to include in a single manuscript and not directly related to the research question at hand.
2. Final analysis uses **CI-ART by 6 months after enrollment as outcome**, while the concept listed a number of possible timepoints (3, 6, 12, and 24 months). In prior analyses for the IeDEA/WHO report, CI-ART was presented graphically as a curve, and thus CI-ART was reported at each of these timepoints. For this analysis, the 6-month timepoint was selected to represent the outcome, timely ART initiation, while allowing sufficient time and opportunity for ART eligibility assessment. The 6-month outcome definition was chosen prior to the start of the analyses.
3. Final analysis uses **site as the unit of analysis**. At the time of concept submission, we were not yet able to select the unit of analysis (e.g. site or country). Because we were still in the process of collecting and verifying national guideline expansion data, we did not know how many patients at how many sites and in how many countries might be eligible for inclusion. We chose site as the analysis unit after data collection was completed and it became clear that a site-level analysis was possible, which avoided the necessity of collapsing patient data by countries, for example. We also considered site the optimal unit of analysis to demonstrate the expected heterogeneity of outcomes across a wide range of diverse facilities.
4. Because site was chosen as the unit of analysis (as opposed to patient), we **used site-level meta-regression** to assess correlates of the outcome, instead of the proposed competing-risk regression.
5. Similarly, because site was chosen as the unit of analysis, **time of country-level guideline expansion was used to generate pre-post comparison periods** rather than as a time-varying covariate, which we considered using in a possible patient-level analysis.
6. The final analysis **does not use log-rank test**, because the analytic approach chosen did not include comparison of survival curves.
7. The final analysis **omits certain proposed stratification variables**, either because
   1. They could not be accessed reliably (pregnancy status, clinical and contextual site characteristics),
   2. They were considered less informative and not a priority in a more focused analysis (World Bank income group), or
   3. They did not make sense in the context of final research design narrowly defining comparison periods (enrollment period [year])
8. Final analysis **does not impute missing enrollment CD4 counts**. Because of the appreciable proportion of patients missing enrollment CD4 counts (see Table 1 in the manuscript) and the potential to introduce bias on a large scale, we decided not to impute missing enrollment CD4 counts.
9. The **number of countries, sites and patients** in the proposed manuscript title and in the Sample section is different, because it was a rough estimate of data that might be eligible for inclusion at the time, before we had final country-level guideline data and before updated IeDEA patient-level datasets became available to us.
10. The final analysis **includes** **data up to 2017**, while the concept proposed to include data only up to 2015. This is because at the time we could not predict exactly how long the analysis would take and how much up-to-date data would become available after the concept was approved.
11. The final analysis **does not include data from West Africa**, which was proposed for inclusion in the concept, because data were only available for patients who initiated ART.

**Concept Proposal Submitted to and Approved by the IeDEA Executive Committee:**

| **Abstract:** | **Background:** Despite improvements in access to ART, increases in the median CD4 cell count at ART initiation have been slow and many HIV-infected people are diagnosed late, experience pre-treatment delays and/or large gaps in continuity of care prior to ART initiation. This results in mortality prior to ART initiation, substantial early mortality on ART, slower CD4 cell count response to ART, more complicated and costly clinical management, and missed opportunities to prevent HIV transmission.  **Objectives:**  Among adults enrolling in HIV care at IeDEA sites between 2004-2015:   1. Assess trends in the proportion of persons who have already progressed beyond ART eligibility, based on CD4 cell count and clinical stage data, by the time of enrollment in HIV care. 2. Assess trends in the proportion lost to the original clinic (LTC) by specific timepoints after enrollment in care, accounting for deaths and ART initiation as competing risks. 3. Assess trends in the proportion of persons initiating ART at the original site of enrollment by specific timepoints after enrollment in care and ART eligibility, accounting for loss to clinic and death as competing risks. 4. Assess the influence of changes in national treatment guidelines on the proportion of patients initiating ART at the original site of enrollment.   **Methods:** Medians and proportions will be used to describe patient status at enrollment in care (objective 1). Using data from sites with consistent pre-ART laboratory and / or visit data, competing risk methods, with Aalen-Johansen estimator, will be employed to calculate the cumulative incidence of LTC and ART initiation in objectives 2 and 3. Analyses will be stratified by key patient, site, and contextual characteristics. We will attempt to incorporate time-updated, country specific information on HIV treatment guidelines, which we are actively compiling, into our analyses. |
| --- | --- |

| **Outline of project** | **Background**  Despite improvements in access to ART across the world [[1](#_ENREF_1)] and increases in the median CD4 cell counts of adults initiating treatment,[[2](#_ENREF_2)] many HIV-infected people in areas with the greatest burden of HIV continue to experience delays and start ART with advanced HIV infection.[[3-5](#_ENREF_3)] In 2012, among persons eligible for treatment in low- and middle-income countries, only 61% were receiving ART (under the 2010 WHO guidelines criteria) or as few as 34% (under the 2013 guidelines criteria). [[1](#_ENREF_1)] Increases in CD4 cell counts at ART initiation have been slow in both low- and high-income countries, with average pooled annual increases ranging from 6 to 11 cells/μL among women, and 5 to 9 cells/μL among men between 2002 and 2009.[[2](#_ENREF_2)] This is especially true in sub-Saharan Africa, where one recent large scale study estimated the median CD4 cell count ART initiation to be 170 cells/µL and increasing slowly at 10 cells/year, implying that it will be another 18 years before only half of patients initiate ART at the current recommended threshold of 350 cells/μL in most SSA countries and 33 years under the expanded WHO guideline threshold of 500 cells/ μL that many countries will be adapting in the coming years. [[6](#_ENREF_6)]  Delays in diagnosis of HIV following infection, pre-treatment delays in care and/or large gaps in continuity of care prior to ART initiation can result in mortality prior to ART initiation, substantial early mortality on ART,[[7](#_ENREF_7)] slower CD4 cell count recovery,[[8](#_ENREF_8)] more complicated and costly clinical management,[[9](#_ENREF_9)] and missed opportunities to prevent HIV transmission.[[10](#_ENREF_10)] Delays in ART initiation among persons eligible for ART result from delayed HIV diagnosis, delayed enrollment in care, or delayed initiation of ART (once enrolled in HIV care). [[5](#_ENREF_5)] This project aims to characterize and pinpoint these delays, including: 1) the extent to which people are already eligible for ART at the time they enroll in care; 2) loss to the original clinic prior to ART initiation; and 3) delays between enrollment, eligibility and ART initiation.  Delays across the HIV continuum may specifically be influenced by patient-, program-, and policy-level factors.[[5](#_ENREF_5)] A variety of clinical and sociodemographic factors have been linked to late presentation to care[[11](#_ENREF_11)] and late treatment initiation[[6](#_ENREF_6), [12](#_ENREF_12), [13](#_ENREF_13)], including patient’s sex, pregnancy status, age, education, family composition, tuberculosis diagnosis, gaps in pre-ART care, treatment eligibility at the time of presentation to care, and source of referral to HIV care.[[6](#_ENREF_6), [14-19](#_ENREF_14)] The timing of ART initiation is also greatly influenced by changes in country-specific HIV treatment guidelines.[[13](#_ENREF_13), [20](#_ENREF_20)] Patient and site-level data from the IeDEA collaboration can be used to better understand the extent of pre-treatment delay among various groups of patients retained in HIV care and to inform strategies for achieving the UNAIDS goal of 90% ART coverage by 2020 (part of the 90-90-90 targets).[[21](#_ENREF_21)] IeDEA data can also be used to examine the extent to which changing treatment guidelines in each country influence key outcomes, such as the proportion initiating ART at the original site.  This concept sheet is part of the IeDEA-WHO collaboration project.  **Primary objective**   1. Assess trends in the proportion of persons who have already progressed beyond ART eligibility, based on CD4 cell count and clinical stage data, by the time of enrollment in HIV care. 2. Assess trends in the proportion lost to the original clinic (LTC) by specific timepoints after enrollment in care, accounting for deaths and ART initiation as competing risks. 3. Assess trends in the proportion of persons initiating ART at the original site of enrollment by specific timepoints after enrollment in care and ART eligibility, accounting for loss to clinic and death as competing risks. 4. Assess the influence of changes in national treatment guidelines on the proportion of patients initiating ART at the original site of enrollment.   We will stratify estimates by:   - Sex - Pregnancy status as documented in the database at enrollment in HIV care and ART initiation - Age at enrollment in HIV care - CD4 cell count at enrollment in HIV care - Clinical stage at enrollment in HIV care - World Bank country income group - IeDEA region and country - Enrollment period - Clinical site characteristics such as urban / rural location, predominant PMTCT service option - Contextual characteristics such as country-specific HIV treatment guidelines, regional / national HIV prevalence, testing and ART coverage, and national PMTCT option   **Secondary objectives**   1. Assess trends in the proportion of patients with the first recorded ART eligibility assessment by specific timepoints after enrollment in care. 2. Correct estimates from primary objectives 2 and 3 for outcome misclassification.[[22](#_ENREF_22)]   **Eligibility criteria**  All adult IeDEA sites are eligible for inclusion. All patients enrolling in HIV care in 2004 or later at 16 years of age or older, regardless of whether they eventually initiated ART, are eligible for inclusion in these analyses.  **Exclusion criteria**   - Patients known not to be ART naïve at enrollment - Patients who transferred into the site regardless of ART status at the time of transfer   **Primary outcomes**   - ART eligibility at enrollment in HIV care: ART eligibility status (yes / no / unknown) based on the CD4 cell count / clinical stage closest to the date of HIV care enrollment within 3 months after enrollment date and no later than 1 week after ART initiation, according to national ART eligibility criteria at the time. If tuberculosis and pregnancy status data are available, they will also be incorporated into the ascertainment of ART eligibility. - ART eligibility: date of the first CD4 cell count and / or clinical stage measurement meeting national ART eligibility criteria at the time. If tuberculosis and pregnancy status data are available, they will also be incorporated into the ascertainment of ART eligibility. - Loss to the original clinic of enrollment (LTC) prior to ART initiation: no clinical visit for 12 months without having ever returned to the clinic or initiated ART, and no documented death or transfer.^[[1]](#footnote-1)^ The time of LTC will be assigned as 90 days following the last known contact with the clinic. - ART initiation: start of a regimen of at least three antiretroviral drugs, in accordance with the IeDEA/WHO collaboration definition. - Time between enrolment in HIV care, ART eligibility, and ART initiation   **Secondary outcomes**   - First ART eligibility assessment: date of first CD4 cell count measurement or clinical stage recorded after enrollment in HIV care. ART initiation will be treated as an explicit indicator of both eligibility assessment and eligibility itself. - Time between enrollment in HIV care and first ART eligibility assessment   Analyses on ART eligibility at enrollment in HIV care and time between ART eligibility and ART initiation (primary objectives 1 and 3) will depend on the availability of time-updated, country-level ART eligibility criteria for 2004-2015, which are still being collected by our team. We have also asked WHO staff for their assistance. In the absence of sufficient national data on ART eligibility criteria, WHO criteria may be used instead.  **Other definitions**   - CD4 cell count / clinical stage at enrollment in HIV care: CD4 cell count measurement / clinical stage closest to the date of HIV care enrollment within 3 months after enrollment date and no later than 1 week after ART initiation.   **Statistical methods**  Descriptive statistics will be used to examine trends in median CD4 cell counts, proportions enrolling in HIV care beyond ART eligibility and initiating ART at the original site of enrollment (primary objectives 1 and 4). We will attempt to impute or extrapolate missing on enrollment CD4 counts using other information in the dataset, such as subsequent CD4 counts prior to ART initiation.  Competing risk methods, with Aalen-Johansen estimator, will be used to calculate cumulative incidence of loss to clinic prior to ART initiation and ART initiation at the original clinic by specific timepoints after enrollment in HIV care (e.g. 3, 6, 12 and 24 months), accounting for respective competing outcomes of death, ART initiation, and loss to clinic. Similar methods will be used for Secondary objectives.  Sensitivity analyses will be completed to correct for outcome misclassification (secondary objective 2) and to assess possible biases related to the dynamic mix of sites contributing data throughout the period under study.  **Sample size considerations**  This study will include all available data from patients with 16 years of age or older enrolled in HIV care at eligible sites. No power calculations have been performed.  **Ethical considerations**  Only anonymized data will be used in this analysis. Data will be obtained from individual IeDEA data coordinating centers, which already have IRB approval for contribution of data to collaborative IeDEA analyses. |
| --- | --- |
| **Target journal:** | **Amendment to specify plans for a peer-reviewed publication. This manuscript will focus on objectives 3 and 4 above.**  Proposed manuscript title: "Cumulative Incidence of ART Initiation at the Original Clinic of Enrolment: An Analysis of IeDEA Network Data from 212 Clinics in 37 Countries, 2004-2015"  Proposed target journals: the Lancet, the Lancet Infectious Diseases, Clinical Infectious Diseases, or AIDS.  Methods and content for the manuscript:  Sample:   - >900,000 ART-naïve, adult patients at 212 IeDEA sites in 37 countries within 6 IeDEA regions (Asia- Pacific, Central Africa, Central/South America and Caribbean, East Africa, North America, Southern Africa). - Data from IeDEA West Africa were not provided in 2015, however they have been provided for 2016 and we proposed to include them as well.   Exclusions:   - <16 years old - not ART-naïve at enrolment - transfer in from another site - no clinic visit record - from sites only providing data for ART initiators - Enrolled <2004 or >2015, or ≤12 months before site database closure   Definitions:   - ART initiation – Start of three-drug combination antiretroviral therapy (cART). - Loss to clinic (LTC) – Lack of clinic contact for 12 months or more since the last visit, without subsequent return within the available follow-up period. Time of LTC for those meeting the definition was set at 3 months after the last visit. - HIV treatment guidelines: We have collected time-updated, country-specific information on HIV treatment guidelines, which will be coded in a standard manner for incorporation into analyses examining the influence of HIV treatment guideline expansion on the likelihood of ART initiation at the original clinic.   Analysis:   - Multivariable competing-risks regression: subhazard ratios (SHR) of ART initiation, adjusted for region, sex, age group, CD4 count at enrolment, and year of enrolment. - Stratified competing-risks models of cumulative incidence of ART initiation (CI-ART), using Aalen-Johansen estimator. Death and LTC will be considered competing events. - Information on HIV treatment guideline changes will be analyzed as time-dependent covariates. - Statistical significance testing via modified log-rank test. - Planned sensitivity analyses restricted analyses to sites which contributed data throughout 2004-2015. - Analyses stratified by CD4 count at enrollment - We will include the median time from enrollment to ART initiation and IQR. - Statistical software: Stata 13.1 (StataCorp, TX, USA). |
| **References** | 1. Joint United Nations Programme on HIV/AIDS. Global Report: UNAIDS Report on the Global AIDS Epidemic 2013. Geneva: UNAIDS, 2013. In.  2. The IeDEA and ART Cohort Collaborations. Immunodeficiency at the start of combination antiretroviral therapy in low-, middle- and high-income countries. *Journal of acquired immune deficiency syndromes (1999)* 2014,**65**:e8-e16.  3. Avila D, Althoff KN, Mugglin C, Wools-Kaloustian K, Koller M, Dabis F*, et al.* Immunodeficiency at the start of combination antiretroviral therapy in low-, middle-, and high-income countries. *Journal of acquired immune deficiency syndromes* 2014,**65**:e8-16.  4. Pati R, Lahuerta M, Elul B, Okamura M, Alvim MF, Schackman B*, et al.* Factors associated with loss to clinic among HIV patients not yet known to be eligible for antiretroviral therapy (ART) in Mozambique. *J Int AIDS Soc* 2013,**16**:18490.  5. Lahuerta M, Ue F, Hoffman S, Elul B, Kulkarni SG, Wu Y*, et al.* The Problem of Late ART Initiation in Sub-Saharan Africa: A Transient Aspect of Scale-up or a Long-term Phenomenon? *J Health Care Poor Underserved* 2013,**24**:359-383.  6. Lahuerta M, Wu Y, Hoffman S, Elul B, Kulkarni SG, Remien RH*, et al.* Advanced HIV Disease at Entry into HIV Care and Initiation of Antiretroviral Therapy During 2006-2011: Findings From Four Sub-Saharan African Countries. *Clin Infect Dis* 2014,**58**:432-441.  7. Lawn SD, Harries AD, Anglaret X, Myer L, Wood R. Early mortality among adults accessing antiretroviral treatment programmes in sub-Saharan Africa. *AIDS* 2008,**22**:1897-1908.  8. Nash D, Katyal M, Brinkhof MW, Keiser O, May M, Hughes R*, et al.* Long-term immunologic response to antiretroviral therapy in low-income countries: a collaborative analysis of prospective studies. *AIDS* 2008,**22**:2291-2302.  9. Krentz HB, Auld MC, Gill MJ. The high cost of medical care for patients who present late (CD4 <200 cells/microL) with HIV infection. *HIV Med* 2004,**5**:93-98.  10. Cohen MS, Chen YQ, McCauley M, Gamble T, Hosseinipour MC, Kumarasamy N*, et al.* Prevention of HIV-1 infection with early antiretroviral therapy. *N Engl J Med* 2012,**365**:493-505.  11. Hoffman S, Wu Y, Lahuerta M, Kulkarni SG, Nuwagaba-Biribonwoha H, Sadr WE*, et al.* Advanced disease at enrollment in HIV care in four sub-Saharan African countries: change from 2006 to 2011 and multilevel predictors in 2011. *AIDS* 2014,**28**:2429-2438.  12. Mutimura E, Addison D, Anastos K, Hoover D, Dusingize JC, Karenzie B*, et al.* Trends in and correlates of CD4+ cell count at antiretroviral therapy initiation after changes in national ART guidelines in Rwanda. *AIDS* 2015,**29**:67-76.  13. Nash D, Tymejczyk O, Gadisa T, Kulkarni SG, Hoffman S, Yigzaw M*, et al.* Factors associated with initiation of antiretroviral therapy in the advanced stages of HIV infection in six Ethiopian HIV clinics, 2012 to 2013. *J Int AIDS Soc* 2016,**19**:20637.  14. Abaynew Y, Deribew A, Deribe K. Factors associated with late presentation to HIV/AIDS care in South Wollo ZoneEthiopia: a case-control study. *AIDS Res Ther* 2011,**8**:8.  15. Drain PK, Losina E, Parker G, Giddy J, Ross D, Katz JN*, et al.* Risk factors for late-stage HIV disease presentation at initial HIV diagnosis in Durban, South Africa. *PLoS One* 2013,**8**:e55305.  16. Geng EH, Hunt PW, Diero LO, Kimaiyo S, Somi GR, Okong P*, et al.* Trends in the clinical characteristics of HIV-infected patients initiating antiretroviral therapy in Kenya, Uganda and Tanzania between 2002 and 2009. *J Int AIDS Soc* 2011,**14**:46.  17. Hoffman S, Wu Y, Lahuerta M, Kulkarni SG, Nuwagaba-Biribonwoha H, Sadr WE*, et al.* Advanced disease at enrollment in HIV care in four sub-Saharan African countries: change from 2006 to 2011 and multilevel predictors in 2011. *AIDS* 2014,**28**:2429-2438.  18. Mulissa Z, Jerene D, Lindtjorn B. Patients present earlier and survival has improved, but pre-ART attrition is high in a six-year HIV cohort data from Ethiopia. *PLoS One* 2010,**5**:e13268.  19. Zango A, Dube K, Kelbert S, Meque I, Cumbe F, Chen PL*, et al.* Determinants of Prevalent HIV Infection and Late HIV Diagnosis among Young Women with Two or More Sexual Partners in Beira, Mozambique. *PLoS One* 2013,**8**:e63427.  20. Mutimura E, Addison D, Anastos K, Hoover DR, Dusingize JC, Karenzi B*, et al.* Trends in and Determinants of CD4+ Cell Count at ART Initiation after Changes in National ART Guidelines in Rwanda. *AIDS* 2015,**29**:67-76.  21. Joint United Nations Programme on HIV/AIDS. 90-90-90: an ambitious treatment target to help end the AIDS epidemic. *Report. Geneva: UNAIDS* 2014.  22. Bakoyannis G, Yiannoutsos CT. Impact of and Correction for Outcome Misclassification in Cumulative Incidence Estimation. *PLoS One* 2015,**10**:e0137454. |

1. During the pre-ART stage of HIV care, compared to shorter definitions, the 12 month definition decreases the risk of misclassification of temporary disengagement as loss to clinic, in the context of varying frequency of follow-up among adult patients. [↑](#footnote-ref-1)
