# Supplementary material for: HIV treatment eligibility expansion and timely antiretroviral treatment initiation following enrollment in HIV care: A metaregression analysis of programmatic data from 22 countries
Source: PLoS Med. 2018 Mar 23;15(3):e1002534. doi: 10.1371/journal.pmed.1002534 (PMC5865713; doi:10.1371/journal.pmed.1002534)
Supplement: S3 Text — (DOCX) [file pmed.1002534.s004.docx]

**Supplement 3: Complete acknowledgments**

**HIV treatment eligibility expansion and timely antiretroviral treatment initiation following enrollment in HIV care – A meta-regression analysis of programmatic data from 22 countries.**

The authors thank patients, providers, and administrative staff at participating IeDEA facilities. We also are grateful for information on ART eligibility expansions contributed by Marion Achieng, Dr. Pedro Cahn, Dr. Morna Cornell, Dr. Andrew Edmonds, Aimee Freeman, Dr. Tsigereda Gadisa, Dr. Anthony Harries, Laura Jefferys, Dr. Marco Luque, Dr. Dominique Mahambou Nsonde, Dr. Paula Mendes Luz, Fernanda Maruri, Dr. Tsitsi Mutasa-Apollo, Dr. Sabin Nsanzimana, Dr. Ng Oon Tek, Dr. Denis Padgett, Boondarika Petersen, Dr. Erik Schouten, Dr. Annette Sohn, Dr. Christelle Twizere, Marco Antonio De Avila Vitoria, Dr. Wong Wing-Wai, and Dr. Marcel Yotebieng.

We also acknowledge the clinics and investigators within each IeDEA region who contributed data to this analysis:

**Asia-Pacific (TAHOD - Adult):**

PS Ly* and V Khol, National Center for HIV/AIDS, Dermatology & STDs, Phnom Penh, Cambodia; FJ Zhang* ‡, HX Zhao and N Han, Beijing Ditan Hospital, Capital Medical University, Beijing, China; MP Lee*, PCK Li, W Lam and YT Chan, Queen Elizabeth Hospital, Hong Kong, China; N Kumarasamy*, S Saghayam and C Ezhilarasi, Chennai Antiviral Research and Treatment Clinical Research Site (CART CRS), YRGCARE Medical Centre, VHS, Chennai, India; S Pujari*, K Joshi , S Gaikwad and A Chitalikar, Institute of Infectious Diseases, Pune, India; TP Merati*, DN Wirawan and F Yuliana, Faculty of Medicine Udayana University & Sanglah Hospital, Bali, Indonesia; E Yunihastuti*, D Imran and A Widhani, Faculty of Medicine Universitas Indonesia - Dr. Cipto Mangunkusumo General Hospital, Jakarta, Indonesia; J Tanuma*, S Oka and T Nishijima, National Center for Global Health and Medicine, Tokyo, Japan; JY Choi*, Na S and JM Kim, Division of Infectious Diseases, Department of Internal Medicine, Yonsei University College of Medicine, Seoul, South Korea; BLH Sim*, YM Gani, and R David, Hospital Sungai Buloh, Sungai Buloh, Malaysia; A Kamarulzaman*, SF Syed Omar, S Ponnampalavanar and I Azwa, University Malaya Medical Centre, Kuala Lumpur, Malaysia; R Ditangco*, E Uy and R Bantique, Research Institute for Tropical Medicine, Manila, Philippines; WW Wong* †, WW Ku and PC Wu, Taipei Veterans General Hospital, Taipei, Taiwan; OT Ng*, PL Lim, LS Lee and Z Ferdous, Tan Tock Seng Hospital, Singapore; A Avihingsanon*, S Gatechompol, P Phanuphak and C Phadungphon, HIV-NAT/Thai Red Cross AIDS Research Centre, Bangkok, Thailand; S Kiertiburanakul*, A Phuphuakrat, L Chumla and N Sanmeema, Faculty of Medicine Ramathibodi Hospital, Mahidol University, Bangkok, Thailand; R Chaiwarith*, T Sirisanthana, W Kotarathititum and J Praparattanapan, Research Institute for Health Sciences, Chiang Mai, Thailand; P Kantipong* and P Kambua, Chiangrai Prachanukroh Hospital, Chiang Rai, Thailand; KV Nguyen*, HV Bui, DTH Nguyen and DT Nguyen, National Hospital for Tropical Diseases, Hanoi, Vietnam; DD Cuong*, NV An and NT Luan, Bach Mai Hospital, Hanoi, Vietnam; AH Sohn*, JL Ross* and B Petersen, TREAT Asia, amfAR - The Foundation for AIDS Research, Bangkok, Thailand; DA Cooper, MG Law*, A Jiamsakul* and R Bijker, The Kirby Institute, UNSW Australia, Sydney, Australia.

(* TAHOD Steering Committee member; † Steering Committee Chair; ‡ co-Chair).

**IeDEA Caribbean, Central, and South America (CCASAnet):**

Fundación Huésped, Argentina: Pedro Cahn, Carina Cesar, Valeria Fink, Omar Sued, Emanuel Dell’Isola, Hector Perez, Jose Valiente, Cleyton Yamamoto

Instituto Nacional de Infectologia-Fiocruz, Brazil: Beatriz Grinsztejn, Valdilea Veloso, Paula Luz, Raquel de Boni, Sandra Cardoso Wagner, Ruth Friedman, Ronaldo Moreira.

Universidade Federal de Minas Gerais, Brazil: Jorge Pinto, Flavia Ferreira, Marcelle Maia.

Universidade Federal de São Paulo, Brazil: Regina Célia de Menezes Succi, Daisy Maria Machado, Aida de Fátima Barbosa Gouvêa

Fundación Arriarán, Chile: Marcelo Wolff, Claudia Cortes, Maria Fernanda Rodriguez, Gladys Allendes

Les Centres GHESKIO, Haiti: Jean William Pape, Vanessa Rouzier, Adias Marcelin, Christian Perodin.

Hospital Escuela Universitario, Honduras: Marco Tulio Luque.

Instituto Hondureño de Seguridad Social, Honduras: Denis Padgett.

Instituto Nacional de Ciencias Médicas y Nutrición Salvador Zubirán, Mexico: Juan Sierra Madero, Brenda Crabtree Ramirez, Paco Belaunzaran, Yanink Caro Vega.

Instituto de Medicina Tropical Alexander von Humboldt, Peru: Eduardo Gotuzzo, Fernando Mejia, Gabriela Carriquiry.

Vanderbilt University Medical Center, USA: Catherine C McGowan, Bryan E Shepherd, Timothy Sterling, Karu Jayathilake, Anna K Person, Peter F Rebeiro, Mark Giganti, Jessica Castilho, Stephany N Duda, Fernanda Maruri, Hilary Vansell.

**Central Africa (CA-IeDEA):**

**Site investigators and cohorts:**

Nimbona Pélagie, ANSS, Burundi; Patrick Gateretse, Jeanine Munezero, Valentin Nitereka, Théodore Niyongabo, Christelle Twizere, Centre National de Reference en Matiere de VIH/SIDA, Burundi; Hélène Bukuru, Thierry Nahimana, CHUK, Burundi; Jérémie Biziragusenyuka, Risase Scholastique Manyundo, HPRC, Burundi; Tabeyang Mbuh, Kinge Thompson Njie, Edmond Tchassem, Bamenda Hospital, Cameroon; Rogers Ajeh, Mark Benwi, Anastase Dzudie, Tsi Kien-Atsu, Akindeh Mbuh, Marc Lionel Ngamani, Victorine Nkome, CRENC & Douala General Hospital, Cameroon; Djenabou Amadou, Eric Ngassam, Eric Walter Pefura Yone, Jamot Hospital, Cameroon; Alice Ndelle Ewanoge, Norbert Fuhngwa, Chris Moki, Denis Nsame Nforniwe, Limbe Regional Hospital, Cameroon; Catherine Akele, Faustin Kitetele, Patricia Lelo, Martine Tabala, Kalembelembe Pediatric Hospital, Democratic Republic of Congo; Emile Wemakoy Okitolonda, Landry Wenzi, Kinshasa School of Public Health, Democratic Republic of Congo; Merlin Diafouka, Martin Herbas Ekat, Dominique Mahambou Nsonde, CTA Brazzaville, Republic of Congo; Adolphe Mafou, CTA Pointe-Noire, Republic of Congo; Fidele Ntarambirwa, Bethsaida Hospital, Rwanda; Yvonne Tuyishimire, Busanza Health Center, Rwanda; Theogene Hakizimana, Gahanga Health Center, Rwanda; Josephine Ayinkamiye, Gikondo Health Center, Rwanda; Sandrine Mukantwali, Kabuga Health Center, Rwanda; Henriette Kayitesi, Olive Uwamahoro, Kicukiro Health Center, Rwanda; Viateur Habumuremyi, Jules Ndumuhire, Masaka Health Center, Rwanda; Joyce Mukamana, Yvette Ndoli, Oliver Uwamahoro, Nyarugunga Health Center, Rwanda; Gallican Kubwimana, Pacifique Mugenzi, Benjamin Muhoza, Athanase Munyaneza, Emmanuel Ndahiro, Diane Nyiransabimana, Jean d'Amour Sinayobye, Vincent Sugira, Rwanda Military Hospital, Rwanda; Chantal Benekigeri, Gilbert Mbaraga, WE-ACTx Health Center, Rwanda.

**Coordinating and Data Centers:**

Adebola Adedimeji, Kathryn Anastos, Madeline Dilorenzo, Lynn Murchison, Jonathan Ross, Albert Einstein College of Medicine, USA; Diane Addison, Ellen Brazier, Heidi Jones, Elizabeth Kelvin, Sarah Kulkarni, Denis Nash, Olga Tymejczyk, Institute for Implementation Science in Population Health, Graduate School of Public Health and Health Policy, City University of New York (CUNY), USA; Batya Elul, Columbia University, USA; Xiatao Cai, Don Hoover, Hae-Young Kim, Chunshan Li, Qiuhu Shi, Data Solutions, USA; Robert Agler, Kathryn Lancaster, Marcel Yotebieng, Ohio State University, USA; Mark Kuniholm, University at Albany, State University of New York, USA; Andrew Edmonds, Angela Parcesepe, University of North Carolina at Chapel Hill, USA; Olivia Keiser, University of Geneva; Stephany Duda; Vanderbilt University School of Medicine, USA; April Kimmel, Virginia Commonwealth University School of Medicine, USA; Margaret McNairy, Weill Cornell Medical Center.

**North America (NA-ACCORD):**

Constance A. Benson and Ronald J. Bosch, AIDS Clinical Trials Group Longitudinal Linked Randomized Trials; Gregory D. Kirk, AIDS Link to the IntraVenous Experience; Stephen Boswell, Kenneth H. Mayer and Chris Grasso, Fenway Health HIV Cohort; Robert S. Hogg, P. Richard Harrigan, Julio SG Montaner, Angela Cescon and Karyn Gabler; HAART Observational Medical Evaluation and Research; Kate Buchacz and John T. Brooks, HIV Outpatient Study; Kelly A. Gebo and Richard D. Moore, HIV Research Network; Richard D. Moore, Johns Hopkins HIV Clinical Cohort; Benigno Rodriguez, John T. Carey Special Immunology Unit Patient Care and Research Database, Case Western; Benigno Rodriguez, Reserve University; Michael A. Horberg, Kaiser Permanente Mid-Atlantic States; Michael J. Silverberg; Kaiser Permanente Northern California; Jennifer E. Thorne, Longitudinal Study of Ocular Complications of AIDS; Charles Rabkin, Multicenter Hemophilia Cohort Study–II; Lisa P. Jacobson and Gypsyamber D’Souza, Multicenter AIDS Cohort Study; Marina B. Klein, Montreal Chest Institute Immunodeficiency Service Cohort; Sean B. Rourke, Anita R. Rachlis, Jason Globerman and Madison Kopansky-Giles, Ontario HIV Treatment Network Cohort Study; Robert F. Hunter-Mellado and Angel M. Mayor, Retrovirus Research Center, Bayamon Puerto Rico; M. John Gill, Southern Alberta Clinic Cohort; Steven G. Deeks and Jeffrey N. Martin, Study of the Consequences of the Protease Inhibitor Era; Pragna Patel and John T. Brooks, Study to Understand the Natural History of HIV/AIDS in the Era of Effective Therapy; Michael S. Saag, Michael J. Mugavero and James Willig, University of Alabama at Birmingham 1917 Clinic Cohort; Joseph J. Eron and Sonia Napravnik, University of North Carolina at Chapel Hill HIV Clinic Cohort; Mari M. Kitahata, Heidi M. Crane and Daniel R. Drozd, University of Washington HIV Cohort; Timothy R. Sterling, David Haas, Peter Rebeiro, Megan Turner, Sally Bebawy and Ben Rogers, Vanderbilt Comprehensive Care Clinic HIV Cohort; Amy C. Justice, Robert Dubrow and David Fiellin, Veterans Aging Cohort Study; Stephen J. Gange and Kathryn Anastos, Women's Interagency HIV Study

**Study administration:**

Executive Committee: Richard D. Moore, Michael S. Saag, Stephen J. Gange, Mari M. Kitahata, Marina Klein, Michael Horberg, Keri N. Althoff, Rosemary G. McKaig and Aimee M. Freeman, Administrative Core: Richard D. Moore, Aimee M. Freeman and Carol Lent, Data Management Core: Mari M. Kitahata, Stephen E. Van Rompaey, Heidi M. Crane, Daniel R. Drozd, Liz Morton, Justin McReynolds and William B. Lober, Epidemiology and Biostatistics Core: Stephen J. Gange, Keri N. Althoff, Alison G. Abraham, Bryan Lau, Jinbing Zhang, Jerry Jing, Cherise Wong, Brenna Hogan, Fidel Desir, Mark Riffon, Elizabeth Humes, and Bin Liu and Bin You.

**East Africa IeDEA:**

**Site investigators and cohorts:**

Diero L, Ayaya S, Sang E, MOI University, AMPATH Plus, Eldoret, Kenya; Bukusi E, Charles Karue Kibaara, Elisheba Mutegi, KEMRI (Kenya Medical Research Institute), Kisumu, Kenya; John Ssali, Mathew Ssemakadde, Masaka Regional Referral Hospital, Masaka, Uganda; Mwebesa Bosco Bwana, Michael Kanyesigye, Mbarara University of Science and Technology (MUST), Mbarara, Uganda; Barbara Castelnuovo; John Michael Matovu, Infectious Diseases Institute (IDI), Mulago, Uganda; Fred Nalugoda, Francis X. Wasswa, Rakai Health Sciences Program, Kalisizo, Uganda; G.R. Somi, Joseph Nondi, NACP (National AIDS Control Program) Dar es Salaam, Tanzania; Rita Elias Lyamuya, Francis Mayanga, Morogoro Regional Hospital, Morogoro, Tanzania; Kapella Ngonyani, Jerome Lwali, Tumbi Regional Hospital, Pwani, Tanzania; Mark Urassa, Denna Michael, Richard Machemba, National Institute for Medical Research (NIMR), Kisesa HDSS, Mwanza, Tanzania; Kara Wools-Kaloustian, Constantin Yiannoutsos, Rachel Vreeman, Beverly Musick, Indiana University School of Medicine, Indiana University, Indianapolis, IN, USA; Batya Elul, Columbia University, New York City, NY, USA; Jennifer Syvertsen, Ohio State University, Columbus, OH, USA; Rami Kantor, Brown University/Miriam Hospital, Providence, RI, USA; Jeffrey Martin, Megan Wenger, Craig Cohen, Jayne Kulzer, University of California, San Francisco, CA, USA; Paula Braitstein, University of Toronto, Toronto, Canada

**IeDEA Southern Africa:**

**Site investigators and cohorts:**

Gary Maartens, Aid for AIDS, South Africa; Michael Vinikoor, Centre for Infectious Disease Research in Zambia (CIDRZ), Zambia; Monique van Lettow, Dignitas, Malawi; Robin Wood, Gugulethu ART Programme, South Africa; Nosisa Sipambo, Harriet Shezi Clinic, South Africa; Frank Tanser, Africa Centre for Health & Population Studies (Hlabisa), South Africa; Andrew Boulle, Khayelitsha ART Programme, South Africa; Geoffrey Fatti, Kheth’Impilo, South Africa; Sam Phiri, Lighthouse Clinic, Malawi; Cleophas Chimbetete, Newlands Clinic, Zimbabwe; Karl Technau, Rahima Moosa Mother and Child Hospital, South Africa; Brian Eley, Red Cross Children's Hospital, South Africa; Josephine Muhairwe, SolidarMed Lesotho; Anna Jores, SolidarMed Mozambique; Cordelia Kunzekwenyika, SolidarMed Zimbabwe, Matthew P Fox, Themba Lethu Clinic, South Africa; Hans Prozesky, Tygerberg Academic Hospital, South Africa.

**Data centers:**

Nina Anderegg, Marie Ballif, Lina Bartels, Julia Bohlius, Frédérique Chammartin, Benedikt Christ, Cam Ha Dao Ostinelli, Matthias Egger, Lukas Fenner, Per von Groote, Andreas Haas, Taghavi Katayoun, Eliane Rohner, Lilian Smith, Adrian Spörri, Gilles Wandeler, Elizabeth Zaniewski, Kathrin Zürcher, Institute of Social and Preventive Medicine, University of Bern, Switzerland; Andrew Boulle, Morna Cornell, Mary-Ann Davies, Victoria Iyun, Leigh Johnson, Mmamapudi Kubjane, Nicola Maxwell, Tshabakwane Nembandona, Patience Nyakato, Ernest Mokotoane, Gem Patten, Michael Schomaker, Priscilla Tsondai, Renee de Waal, School of Public Health and Family Medicine, University of Cape Town, South Africa.
